# PROTECT Trial: A cluster-randomized study with hydroxychloroquine versus observational support for prevention or early-phase treatment of Coronavirus disease (COVID-19): A structured summary of a study protocol for a randomized controlled trial

**DOI:** 10.1186/s13063-020-04527-4

**Published:** 2020-07-31

**Authors:** Oriana Nanni, Pierluigi Viale, Bernadette Vertogen, Claudia Lilli, Chiara Zingaretti, Caterina Donati, Carla Masini, Manuela Monti, Patrizia Serra, Roberto Vespignani, Veruska Grossi, Annibale Biggeri, Emanuela Scarpi, Francesca Galardi, Lucia Bertoni, Americo Colamartini, Fabio Falcini, Mattia Altini, Ilaria Massa, Raffaella Gaggeri, Giovanni Martinelli

**Affiliations:** 1 Istituto Scientifico Romagnolo per lo Studio e la Cura dei Tumori (IRST) IRCCS, Meldola, Italy; 2 Dipartimento di Scienze Mediche e Chirurgiche, Università di Bologna, Bologna, Italy; 3 Dipartimento di Statistica, Informatica, Applicazioni “G. Parenti” (DISIA), Università degli studi Firenze, Florence, Italy

**Keywords:** COVID-19, Randomized controlled clinical trial, Protocol, Hydroxychloroquine, Prophylaxis, Prevention, Early treatment, Asymptomatic, Paucisymptomatic

## Abstract

**Objectives:**

Hydroxychloroquine has shown to have antiviral activity *in vitro* against coronaviruses, specifically SARS-CoV-2. It is believed to block virus infection by increasing endosomal pH required for virus cell fusion and glycosylation of viral surface proteins. In addition to its antiviral activity, hydroxychloroquine has an immune-modulating activity that may synergistically enhance its antiviral effect *in vivo,* making it a potentially promising drug for the prevention and the cure of SARS-CoV-19. However, randomized controlled trials are needed to assess whether it can be used safely to treat COVID-19 patients or to prevent infection. The main objective of the present study is to evaluate the efficacy of hydroxychloroquine for (I) the prevention of COVID-19 or related symptoms in SARS-CoV-2-exposed subjects, such as as household members/contacts of COVID-19 patients and (II) the treatment of early-phase asymptomatic or paucisymptomatic COVID-19 patients.

**Trial design:**

This is a controlled, open label, cluster-randomized, superiority trial with parallel group design. Subjects will be randomized either to receive hydroxychloroquine or to observation (2:1).

**Participants:**

SARS-CoV-2-exposed subjects, including household members and/or contacts of COVID-19 patients and healthcare professionals (Group 1) or patients with COVID-19 (positive PCR test on a rhinopharyngeal or oropharyngeal swab for SARS-CoV-2), asymptomatic or paucisymptomatic in home situations who are not undergoing treatment with any anti COVID-19 medication (Group 2), will be enrolled. Paucisymptomatic patients are defined as patients with a low number of mild symptoms. All subjects must be aged ≥18 years, male or female, must be willing and able to give informed consent and must not have any contraindications to take hydroxychloroquine (intolerance or previous toxicity for hydroxychloroquine/chloroquine, bradycardia or reduction in heart rhythm with arrhythmia, ischemic heart disease, retinopathy, congestive heart failure with use of diuretics, favism or glucose-6-phosphate dehydrogenase (G6PD) deficiency, diabetes type 1, major comorbidities such as advanced chronic kidney disease or dialysis therapy, known history of ventricular arrhythmia, any oncologic/hematologic malignancy, severe neurological and mental illness, current use of medications with known significant drug-drug interactions, and known prolonged QT syndrome or current use of drugs with known QT prolongation).

The study is monocentric and will be conducted at Istituto Scientifico Romagnolo per lo Studio e la Cura dei Tumori (IRST) IRCCS. Subjects will be enrolled from a large epidemic region (North-Central Italy). The Public Health Departments of several Italian regions will collaborate by identifying potentially eligible subjects.

**Intervention and comparator:**

The participants will be randomized (2:1 randomization) to receive either hydroxychloroquine (Arm A) or to Observation (Arm B). Hydroxychloroquine will be administered with the following schedule:

Group1: A loading dose hydroxychloroquine 400 mg twice daily on day 1, followed by a weekly dose of hydroxychloroquine 200 mg twice daily on days 8, 15 and 22, for a total of one month of treatment.

Group 2: A loading dose hydroxychloroquine 400 mg twice daily on day 1 followed by 200 mg twice daily for a total of 5-7 days.

The comparator in this trial is observation given that currently neither treatment is administered to asymptomatic or paucisymptomatic subjects, nor prophylaxis is available for contacts.

Hydroxychloroquine will be shipped to subjects within 24 hours of randomization. Given the extraordinary nature of the COVID-19 pandemic, only telephonic interviews will be carried out and electronic Patient Reported Outcomes (ePRO) completed. During treatment, each subject will be contacted every other day for the first week and weekly thereafter (Group 2) or weekly (Group 1) by a study physician to assess early onset of any COVID-19 symptom or any adverse reaction to hydroxychloroquine and to check subject compliance. Furthermore, all subjects will receive periodic ePROs which may be completed through smartphone or tablets to record drug self-administration and onset of any symptom or adverse event. All subjects will be followed up for a total of 6 months by periodic telephonic interviews and ePROs.

**Main outcomes:**

The primary endpoint/outcome measure for this trial is: for Group 1, the proportion of subjects who become symptomatic and/or swab-positive in each arm within one month of randomization; for Group 2, the proportion of subjects who become swab-negative in each arm within 14 days of randomization.

**Randomization:**

All household members and/or contacts of each COVID-19 index case, and the COVID-19 patient himself/herself, fulfilling all inclusion criteria will be grouped into a single cluster and this cluster will be randomized (2:1) to either arm A or arm B. Information on each subject will be recorded in specific data records.

Randomization lists will be stratified according to the following factors regarding COVID-19 index cases:

1. COVID-19 risk level on the basis of province of residence (high *vs.* low/intermediate);

2. Index case is a healthcare professional (yes *vs.*no)

3. Index case with COVID-19 treatment (yes *vs.* no)

An independent statistician not otherwise involved in the trial will generate the allocation sequence, and COVID-19 response teams will be unaware of the allocation of clusters.

Randomization will be performed through an interactive web-based electronic data-capturing database.

An Independent Data Monitoring Committee has been established.

**Blinding (masking):**

This study is open label.

**Numbers to be randomized (sample size):**

For Group 1, a sample size of about 2000 SARS-CoV-2-exposed subjects such as household members and/or contacts of COVID-19 patients will take part in the study. Assuming around 1.5-2.0 asymptomatic household members and/or contacts for each COVID-19 patient, we expect to identify approximately 1000-1300 COVID-19 index cases to be randomized. An interim analysis on efficacy is planned using standard alpha-spending function.

For Group 2, sufficient power for primary objective (negative swab within 14 days of randomization) will be reached given a sample size of 300 asymptomatic or paucisymptomatic COVID-19 subjects in home situations not treated for COVID-19 (25%-30% of about 1000-1300 expected index cases). Since up to date reduced evidence about COVID-19 infection epidemiology, the continuous update of diagnostic and therapeutic approaches, the sample size estimation could be updated after a one third of population will be recruited and eventually modified according to a substantial protocol amendment. An interim analysis at 100 enrolled COVID-19 patients is planned.

We have planned a Generalized Estimating Equation analysis, which is more efficient than a cluster level analysis, to take advantage of subject-specific covariates. The above reported sample size analysis is therefore to be considered conservative.

**Trial Status:**

The current version of the PROTECT trial protocol is ‘Final version, 15 April 2020’.

The study started on 9^th^ May 2020. The first patient was enrolled on 14^th^ May 2020. Recruitment is expected to last through September 2020.

**Trial registration:**

The PROTECT trial is registered in the EudraCT database (no. 2020-001501-24) and in ClinicalTrials.gov (NCT04363827), date of registration 24 April 2020.

**Full protocol:**

The full PROTECT protocol is attached as an additional file, accessible from the Trials website (Additional file 1). In the interests of expediting dissemination of this material, the familiar formatting has been eliminated; this Letter serves as a summary of the key elements of the full protocol (Protocol final version, 15^th^ April 2020). The study protocol has been reported in accordance with Standard Protocol Items: Recommendations for Clinical Interventional Trials (SPIRIT) guidelines (Additional file 2).

## Supplementary information

**Additional file 1.** Full Study Protocol.

**Additional file 2.** SPIRIT 2013 Checklist: Recommended items to address in a clinical trial protocol and related documents.

## Data Availability

Not applicable.

