# Supplementary material for: PROTECT Trial: A cluster-randomized study with hydroxychloroquine versus observational support for prevention or early-phase treatment of Coronavirus disease (COVID-19): A structured summary of a study protocol for a randomized controlled trial
Source: Trials. 2020 Jul 31;21:689. doi: 10.1186/s13063-020-04527-4 (PMC7393246; doi:10.1186/s13063-020-04527-4)
Supplement: Supplementary file 1 — Additional file 1. Full Study Protocol. [file 13063_2020_4527_MOESM1_ESM.pdf]

SERVIZIO SANITARIO REGIONALE
EMILIA-ROMAGNA
Istituto Scientifico Romagnolo per lo Studio e la Cura dei Tumori
Istituto di Ricovero e Cura a Carattere Scientifico

ISTITUTO SCIENTIFICO ROMAGNOLO PER LO STUDIO E LA CURA DEI TUMORI

PROTECT: A randomized study with Hydroxychloroquine versus observational support for prevention or early phase treatment of Coronavirus disease (COVID-19)

Protocol Code:

IRST100.47

IRST Identifier Code: L2P2266

Eudract number: **2020-001501-24**

Date and Version No: 15.04.2020 - Final Version

Short title / Acronym:

PROTECT

PROTECT

Chief Investigators: **Prof. Pierluigi Viale,**
University of Bologna
Director "Dipartimento di Scienze Mediche e Chirurgiche".

Prof Giovanni Martinelli,
Istituto Scientifico Romagnolo per lo Studio e la Cura dei Tumori - IRST IRCCS
Scientific Director

Confidentiality Statement
This document contains confidential information that must not be disclosed to anyone other than the Sponsor/Promoter, the Investigator's Team, IRST IRCCS, regulatory authorities, and members of the Ethics Committee.

KEY TRIAL CONTACTS

**Chief Investigators**	**Pierluigi Viale,**<br>University of Bologna<br>Director Dipartimento di Scienze Mediche e Chirurgiche<br>**Giovanni Martinelli,**<br>IRST-IRCCS della Romagna<br>Scientific Director
**Scientific Committee**	Pierluigi Viale, University of Bologna<br>Giovanni Martinelli, Scientific Director, IRST IRCCS<br>Oriana Nanni, Director Biostatistics and clinical trials unit (UBSC), IRST IRCCS<br>Vittorio Sambri, Director UOC Microbiologia, AUSL della Romagna<br>Fabio Falcini, Ugo De Giorgi, Gerardo Musuraca, “Dipartimento di oncologia ed ematologia clinica e sperimentale”, IRST IRCCS<br>Mattia Altini, Medical Director, IRST IRCCS<br>Annibale Biggeri, University of Florence<br>Zeno Bisoffi , Istituto Sacro Cuore Negrar IRCCS Verona
**Key Protocol Contributors**	Carla Masini, Caterina Donati, Pharmacy<br>Bernadette Vertogen, Chiara Zingaretti, Patrizia Serra,<br>Manuela Monti, Claudia Lilli, Emanuela Scarpi, UBSC<br>Roberto Vespignani, IT Unit<br>Lucia Bertoni, Health Management Office<br>Raffaella Gaggeri- Pharmacovigilance
**Independent Data Monitoring Committee**	Antonello Di Paolo, University of Pisa<br>Alessandra Gennari, University Piemonte Orientale<br>Federico Pea, University of Udine
**Sponsor/Promoter**	Istituto Scientifico Romagnolo per lo Studio e la Cura dei Tumori – IRST IRCCS<br>Via Piero Maroncelli, 40/42<br>47014 Meldola (FC)<br>Italy
**Regional Involved Operating Units**	Servizio Igiene Pubblica, Regione Emilia Romagna<br>Medici di Medicina Generale, Regione Emilia Romagna<br>Laboratori di Microbiologia, Regione Emilia Romagna<br>Unità di Malattie Infettive, Regione Emilia Romagna
**Coordinating Center**	Biostatistics and clinical trials unit (UBSC),<br>Istituto Scientifico Romagnolo per lo Studio e la Cura dei Tumori - IRST IRCCS<br>Via Piero Maroncelli, 40/42<br>47014 Meldola (FC)

	Telephone + 390544285813 Fax +390544285330<br>e-mail address: cc.ubsc@irst.emr.it
**Statistician**	Oriana Nanni<br>Biostatistics and clinical trials unit (UBSC), IRST IRCCS<br>Istituto Scientifico Romagnolo per lo Studio e la Cura dei Tumori - IRST IRCCS<br>Via Piero Maroncelli, 40/42<br>47014 Meldola (FC)<br>Telephone + 390543739266
**Trials pharmacist**	Caterina Donati<br>Pharmacy<br>Istituto Scientifico Romagnolo per lo Studio e la Cura dei Tumori - IRST IRCCS<br>Via Piero Maroncelli, 40/42<br>47014 Meldola (FC)<br>Telephone + 390543739289
**Emergency contact**	Pierluigi Viale pierluigi.viale@unibo.it tel. 3498297428<br>Giovanni Martinelli giovanni.martinelli@irst.emr.it tel. 0543739480 3487704650<br>Oriana Nanni oriana.nanni@irst.emr.it tel. 0543739266

SERVIZIO SANITARIO REGIONALE
EMILIA-ROMAGNA
Istituto Scientifico Romagnolo per lo Studio e la Cura dei Tumori
Istituto di Ricovero e Cura a Carattere Scientifico

ISTITUTO SCIENTIFICO ROMAGNOLO PER LO STUDIO E LA CURA DEI TUMORI

PROTOCOL SIGNATURE PAGE

Protect: A randomized study with Hydroxychloroquine versus observational support for prevention or early phase treatment of Coronavirus disease (COVID-19).

EudraCT number: 2020-001501-24

The undersigned agree and confirm that:

The following protocol has been agreed and accepted and the Chief Investigator agrees to conduct the trial in compliance with the approved protocol and will adhere to the principles outlined in the ICH GCP guidelines, REGULATION (EU) No 536/2014, and any subsequent amendments of the clinical trial regulation,Sponsor/Promoter SOP's and other regulatory requirements as amended.

The confidential information contained in this document will not be used for any other purpose other than the evaluation or conduct of the clinical investigation without the prior written consent of the Sponsor/Promoter.

The findings of the study will be made publically available through publication or other dissemination tools without any unnecessary delay and that an honest accurate and transparent account of the study will be given; and any discrepancies from the study as planned in this protocol will be explained.

Prof. Pierluigi Viale<br>Chief Investigator	______________________________<br>Signature	____________________<br>Date
Prof. Giovanni Martinelli<br>Chief Investigator	______________________________<br>Signature	____________________<br>Date
Oriana Nanni<br>Trial Statistician	______________________________<br>Signature	____________________<br>Date

By signing this document, I am confirming that I have read the protocol for the above study and I agree to conduct the study in compliance with the protocol and ICH GCP

______________________<br>Principal Investigator	______________________________<br>Signature	____________________<br>Date

SUMMARY

**Title**	Protect: A randomized study with Hydroxychloroquine versus observational support for prevention and early phase treatment of Coronavirus disease (COVID-19).
**Short Title/ Acronym**	PROTECT
**Protocol Code**	IRST100.47<br>Identifier Code: L2P2266.
**Phase**	Clinical phase II study
**Study Design**	This is a Italian, superiority, open label cluster-randomised, interventional clinical trial aimed at assessing whether the treatment with Hydroxychloroquine can reduce the percentage of symptomatic subjects compared to observation only in household members/contacts of COVID-19 patients (Group 1) and if the treatment with Hydroxychloroquine could be introduced in early phase COVID-19 population (Group 2).<br>The participants will be randomised to receive either:<br>Arm A) hydroxychloroquine vs<br>Arm B) Observation (2:1 randomisation).
**Background and Rationale for study**	Novel pneumonia caused by a previously unknown pathogen emerged in Wuhan. The pathogen was soon identified as a novel coronavirus (2019-nCoV), which is closely related to severe acute respiratory syndrome CoV. Currently, there is neither specific treatment nor prophylaxis against the new virus SARS-CoV-2 in healthy subjects. Therefore, the current emergency situation warrants for the urgent development of potential strategies to protect people at high risk of infection, particularly cohabitants of diagnosed COVID-19 patients.<br>Antiviral drugs administered shortly after symptom onset can reduce the spread of infection by reducing viral shedding in the respiratory secretions of patients (SARS-CoV-2 viral load in sputum peaks at around 5-6 days after symptom onset and lasts up to 14 days), and targeted prophylactic treatment of contacts could reduce their risk of becoming infected. Implementing antiviral treatment (particularly for COVID19 patients, untreated, and only observed during at-home quarantine) and prophylaxis requires adequate drug availability, and the safety of the procedure must be high.<br>Hydroxychloroquine is a drug that is available for chemoprophylaxis and treatment of malaria. It is registered and used as a disease-modifying antirheumatic drug. It has a long history, is safe and well-tolerated at typical doses. Moreover, hydroxychloroquine show to have antiviral activity in vitro against coronaviruses and specifically SARS-CoV-2. Hydroxychloroquine may be a promising drug for the prevention and the cure of SARS-CoV-19. Ingested days before the virus is introduced to the body, it will reach the serum concentration ranging the EC50 values of 6.25 and 5.85 micromolar at 24 and 48 hours. The drug can accumulate at high levels in lung tissue. Based on physiological pharmacokinetic models studies and by in vitro data results, the possibility to reach high

	concentrations of hydroxychloroquine in lung fluid was demonstrated. A single dose of hydroxychloroquine at 800 mg may provide a lung tissue concentration that is more than twenty times higher than EC50 values necessary to inhibit SARS-CoV-2 in the lung on day 1. It is plausible that a single dose of 400 mg or even 200 mg can provide adequate lung tissue concentration to inhibit SAR-CoV-2. Since the half-life after a single dose of 200 mg is 22 days, a single dose every three weeks should be sufficient for the prevention of SARS-CoV-2 induced lung damage. The blood or sinus concentrations may not be enough to eradicate the virus; however, prevention of lung damage may convert this deadly infection into an upper respiratory infection. Several drugs, such as chloroquine have been used in patients with SARS or MERS. Standard assays were carried out to measure the effects of this compound on the cytotoxicity, virus yield and infection rates of 2019-nCoVs. Chloroquine blocked virus infection at low-micromolar concentration and showed high selectivity index. Chloroquine and Hydroxychloroquine is known to block virus infection by increasing endosomal pH required for virus cell fusion and glycosylation of viral surface proteins. Besides its antiviral activity, chloroquine has an immune-modulating activity, which may synergistically enhance its antiviral effect in vivo. Several clinical trials with Hydroxychloroquine treatment for COVID-19 are ongoing in China (NCT04261517 and NCT0437693). From the first study preliminary data are available, but they are not conclusive because of the small sample size.<br>As the COVID-19 spreads, efforts are made to reduce transmission via standard public health interventions based on isolation of cases and tracing contacts, but such strategy could contribute to reducing the overall size of an outbreak, but will still not be sufficient to achieve outbreak control of COVID-19 when the basic reproduction number (R_0)is higher than 1.5 or the proportion of contacts traced is lower than 80%. Another assumption is that isolation of cases is 100% effective in stopping transmission, yet home confinement of infected individuals and contacts is challenging, efficacy is variable, and rigorous tracking involves a considerable amount of public health resources.<br>Therefore, our institute is planning a controlled cluster-randomised study with Hydroxychloroquine versus observation for prevention of Coronavirus disease (COVID-19) in SARS-CoV-2-exposed subjects with an intermediate-high risk of infection and for treatment of early phase COVID-19 patients.
**Timelines**	Study start (FPFV): April 2020<br>Recruitment end (LPFV): July 2020<br>Treatment period end date (LP off treatment): August 2020<br>Follow-up period end date (LPLV): January 2021<br>The overall study duration will be 10 months; 3 months for subjects enrollment, and 1 month of treatment and further 6 months of follow-up

**Study population**	**Group 1**: SARS-CoV-2-exposed subjects, as household members/contacts of COVID-19 patients<br>**Group 2**: Patients with COVID-19 asymptomatic o paucisymptomatic in home situation.	
**Objectives**	Objectives	Outcomes
**Primary Objectives**	Group 1: Prevention of COVID-19 or related symptoms in household members/contacts of COVID-19 patients within one month from randomization<br>Group 2: Efficacy of Hydroxychloroquine in early phase COVID-19 within 14 days from randomization	Group 1: The primary endpoint/outcome measure is the proportion of subjects of Group 1 who become symptomatic and/or swab positive in each arm within 1 month from randomization.<br>Group 2: The primary endpoint/outcome measure is the proportion of subjects of Group 2 who become swab negative in each arm within 14 days from randomization.
**Main secondary Objectives**	1. To compare the efficacy of prophylaxis with Hydroxychloroquine in prevention of COVID-19 infection (swab positive) in a population of SARS-CoV-2-exposed subjects composed by household members/contacts of COVID-19 patients respect to observation only.<br>2. To assess the efficacy of prophylaxis with Hydroxychloroquine in subgroup population identified by stratification factors, class of age and gender.<br>3. To assess the efficacy of Hydroxychloroquine in early phase COVID-19 patients within 14 days from randomization in subgroup population identified by stratification factors, class of age and gender<br>4. To assess the efficacy of Hydroxychloroquine in early phase COVID-19 patients within 1 month from randomization in overall population and in subgroup population identified by stratification factors, class of age and	1. The proportion of subjects with positive swab in randomized population of SARS-CoV-2-exposed subjects (Group 1) within 1 month from randomization in both arms.<br>2. The proportion of subjects of Group 1 who become symptomatic in each arm within 1 month from randomization, in subgroup population identified by stratification factors, class of age and gender.<br>3. The proportion of subjects of Group 2 who become swab negative in each arm within 14 days from randomization, in subgroup population identified by stratification factors, class of age and gender.<br>4. The proportion of subjects of Group 2 who become swab negative in each arm within 1 month from randomization in overall population and in subgroup population identified by stratification factors, class of age

	gender.<br>5. To evaluate, treatment toxicity of hydroxychloroquine in SARS-CoV-2-exposed subjects population and COVID-19 patients<br>6. To evaluate Quality of Life (EQ-5D-5L) from SARS-CoV-2-exposed subjects population and COVID-19 patients	and gender.<br>5. Absolute and relative frequencies of Serious Adverse Events (CTCAE version 5.0) in both arms for the Group 1 and Group 2.<br>6. Variation in Quality of Life scores in different time points (weekly) respect to baseline values in both Group 1 and Group 2 populations.
**Diagnosis and Main Inclusion Criteria**	Inclusion criteria:<br>1. Male or Female, aged >= 18 years<br>2. SARS-CoV-2-exposed subjects, as households and/or contacts of COVID-19 patients (Group 1). In this group are included health care professionals in contact with COVID-19 patients.<br>or<br>3. Patients with COVID-19, asymptomatic or paucisymptomatic in home situation who are not in treatment with any anti COVID-19 medication (Group 2)<br>4. Absence of any COVID-19 symptom in last week before randomization (fever >37.5°C, cough, dyspnea) (only for group 1 subjects)<br>5. Paracetamol treatment is accepted only for group 2.<br>6. Participant is willing and able to give informed consent for participation in the study (either recorded during a telephonic interview or signed in person) and agrees with the study and its conduct.	
**Study treatment**	The participants will be cluster-randomised (2:1 randomisation) to receive either:<br>**Arm A) Hydroxychloroquine**<br>**Group1:** A loading dose Hydroxychloroquine 400 mg twice daily at day 1, followed by a weekly dose of Hydroxychloroquine 200 mg twice daily on days 8, 15 and 22, covering a total of 1 month of treatment.<br>**Group 2:** A loading dose Hydroxychloroquine 400 mg twice daily at day 1 followed by 200 mg twice daily for a total of at least 5-7 days according to clinical evolution (Region of Emilia Romagna Guideline).<br>**Arm B) Observation.**	
**Sample size**	**For Group 1**:<br>A sample size of about 2000 SARS-CoV-2-exposed subjects as household members and/or contacts of COVID-19 patients will participate into the study. Assuming about 1.5-2.0 asymptomatic household members and/or contacts for each COVID-19 patient, we expected to identify approximately 1000-1300 COVID-19 index cases.<br>**For Group 2:**<br>Sufficient power for primary objective (negative swab within 14 days from randomization) will be reached, given a sample size of 300 COVID-19 subjects asymptomatic or paucisymptomatic in home situation not treated for COVID-19 (25-30% of about 1000-1300 expected case index COVID-19 patients).	

	Since up to date reduced evidences about COVID-19 infection epidemiology, the continuous update of diagnostic and therapeutic approaches, the sample size estimation could be updated after a one third of population will be recruited and eventually modified according a substantial protocol amendment.
**Statistical analysis**	We planned a Generalized Estimating Equation analysis to get advantage of subject-specific covariates. This is therefore more efficient than a cluster level analysis. The above reported sample size analysis is therefore to be considered conservative.
**Significance and innovation**	Currently few informations are available of COVID-19 asymptomatic carriers. Few data are available on prevention and prophylaxis with short exposure, active agents such as Hydroxychloroquine on reducing COVID-19 related symptoms, recovery, progression to use intensive care units/procedures. This protocol could give us additional information on several aspects related to COVID-19 spread prevention and rapid identification of newly symptomatic outcomes, reducing hospitalization, and favoring outcome and care.

ABBREVIATIONS

AE	Adverse event
AR	Adverse reaction
CC	Coordinating centre
CI	Confidence interval
CI	Chief Investigator
CRA	Clinical Research Associate (Monitor)
CRF	Case Report Form
CRO	Contract Research Organisation
CT	Clinical Trials
CTC	Common toxicity criteria
FPFV	First Patient First Visit
GCP	Good Clinical Practice
GP	General Practitioner
HCQ	Hydroxychloroquine
IB	Investigator's Brochure
ICF	Informed Consent Form
ICH	International Conference of Harmonisation
IDMC	Independent Data Monitoring Committee
IEC	Independent Ethics Committee
IRB	Independent Review Board
LPFV	Last Patient First Visit
LP	Last Patient
LPLV	Last Patient Last Visit
PI	Principal Investigator
PIL	Participant/ Patient Information Leaflet
PR	Partial response
SAE	Serious Adverse Event
SAR	Serious Adverse Reaction
SOP	Standard Operating Procedure
SUSAR	Suspected Unexpected Serious Adverse Reactions
WHO	World Health Organization

STUDY SCHEMA

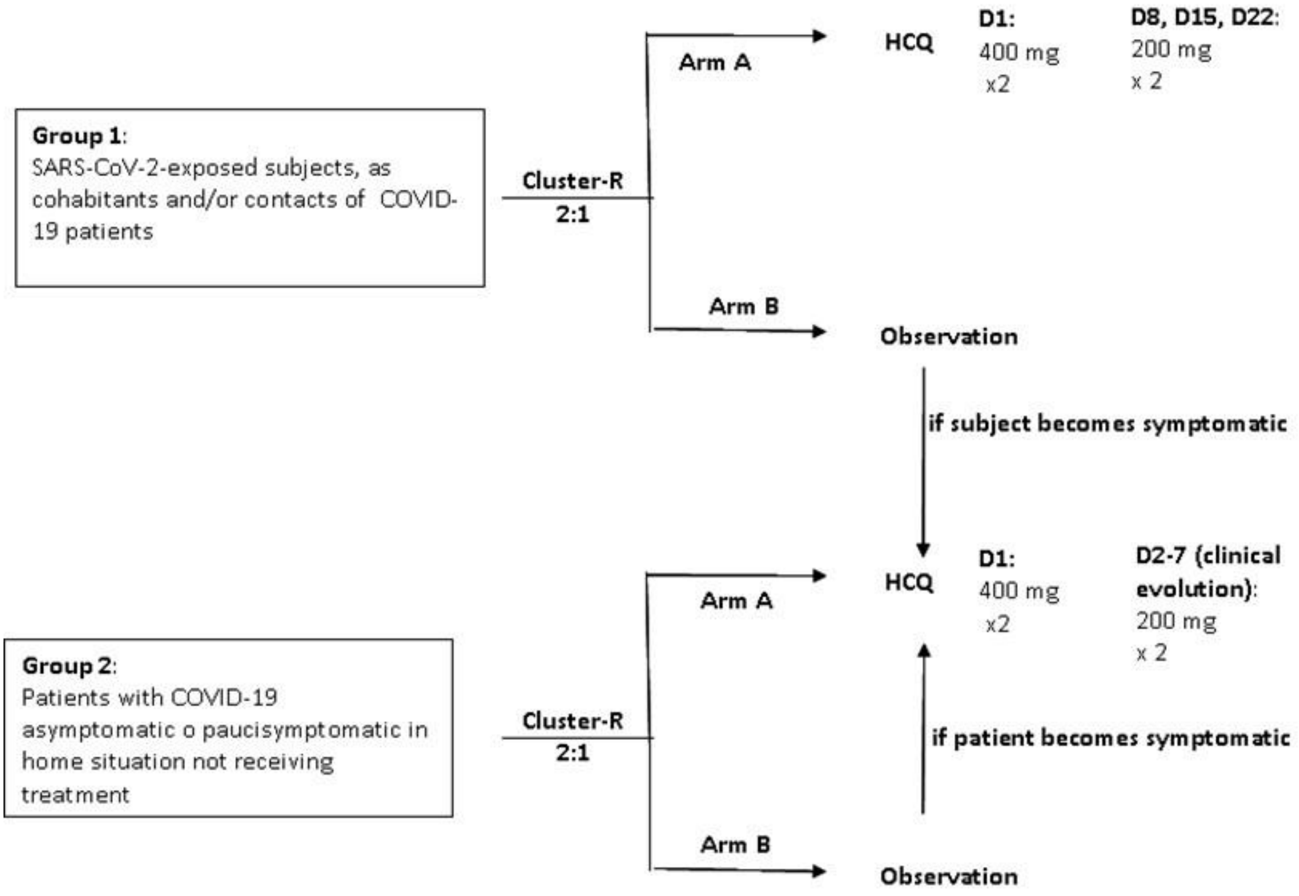

TABLE OF CONTENTS

KEY TRIAL CONTACTS 2
PROTOCOL SIGNATURE PAGE 4
SUMMARY 4
ABBREVIATIONS 10
STUDY SCHEMA 11

1. INTRODUCTION 15
1.1 Background about SARS-CoV-2 15
1.2 Potential Investigational agent (s) against COVID-19 infection. 20
1.3 Investigational agent (s) 24
1.4 Clinical Data to Date 24
1.5 Rationale and Risk/Benefits 25

2. OBJECTIVES AND OUTCOME MEASURES/ENDPOINTS 26
2.1 Primary Objectives 26
2.2 Secondary Objective 26
2.3 Explorative Objective 26
2.4 Primary endpoint/outcome 27
2.5 Secondary endpoints/outcomes 27

3. STUDY DESIGN 28
3.1 Summary of Trial Design 28
3.2 End of trial definition 29

4. STUDY POPULATION 29
4.1 Inclusion Criteria 29
4.2 Exclusion Criteria 30

5. STUDY PROCEDURES 31
5.1 Identification of eligible subjects 31
5.2 Eligibility check 31
5.3 Informed Consent 32
5.3 Registration and Assignment of Subject ID 33
5.4 Screening and Eligibility Assessments 33
5.5 Randomisation 34
5.6 Assessments during treatment/observation 34
5.7 Follow-up assessments 35
5.8 Discontinuation/ Withdrawal of Participants from Study Treatment 36
5.9 Source Data 37

6. STUDY TREATMENT 37
6.1 Description of Study Treatment 38
6.2 Supply of study treatment 38
6.3 Compliance with Study Treatment (if applicable) 39
6.4 Accountability of the Study Treatment 39
6.5 Concomitant Medication 39

7. DOSING DELAYS / DOSE MODIFICATIONS 40
7.1 Study treatment modifications 40

8. BIOMARKER / CORRELATIVE / SPECIAL STUDIES 40

9. SAFETY REPORTING 41
9.1 Definitions 42
9.2 Reporting Procedures for All Adverse Events 45
9.3 Reporting Procedures for Serious Adverse Events 46

10. STATISTICAL CONSIDERATIONS 47
10.1 Sample Size, Accrual Rate and Study Duration 47
10.2 Stratification Factors 55
10.3 Statistical analysis 55
10.4 Reporting and Exclusions 55

11. ETHICAL ASPECTS 55
11.1 Declaration of Helsinki 55
11.2 ICH Guidelines for Good Clinical Practice 55
11.3 Independent Ethical Committee (IEC) 56
11.4 Informed Consent 56
11.5 Patient data protection 56

12. DATA COLLECTION 57

13. STUDY MONITORING 57
13.1 Site Set-up and Initiation 58
13.2 Study Monitoring 58
13.3 Central Monitoring 59
13.4 Independent Data Monitoring Committee 59
13.5 Audit and Inspection 59

14. ADMINISTRATIVE REGULATIONS 59
14.1 Curriculum vitae 59
14.2 Secrecy agreement 60

14.3 Availability and Retention of Investigational Records 60
14.4 Insurance 60

15. OWNERSHIP OF DATA AND USE OF THE STUDY RESULTS 60

16. PUBLICATION POLICY 60

17. PROTOCOL AMENDMENTS 61

18. REFERENCES 61
APPENDIX ANCI Common Terminology Criteria for AE 65
APPENDIX B Schedule of procedures 65
APPENDIX C World Medical Association Declaration of Helsinki 67

1. INTRODUCTION

This document is a protocol for a human research study. This clinical trial is to be conducted in compliance with the protocol, with the REGULATION (EU) No 536/2014, with the principles of ICH Good Clinical Practice, and institutional research policies and procedures.

1.1 Background about SARS-CoV-2

Novel pneumonia caused by a previously unknown pathogen emerged in Wuhan. The pathogen was soon identified as a novel coronavirus (2019-nCoV), which is closely related to severe acute respiratory syndrome CoV. Currently, there is neither specific treatment nor prophylaxis against the new virus SARS-CoV-2. Therefore, identifying effective antiviral agents to combat and prevent the disease is urgently needed [1].

The novel coronavirus known as SARS-CoV-2, with a zoonotic origin, is the agent of Coronavirus Disease 2019 (COVID-19), which is the cause of the world epidemic, with a reported fatality of about 2-3% and a incubation period of 2-14 days [2][3].

The disease has a preponderance for adults, especially the elderly and those with comorbidities. Patients aged >80 years are overrepresented among those diagnosed with the disease (32% of those diagnosed, despite making up 18% of the affected population).

At the moment no vaccine or treatments are available, although tocilizumab has shown interesting data in China and is also being used in our country for severe cases with related cytokine-associated symptoms [4]. No prophylaxis strategy has, at the moment, found a consensus, but hydroxychloroquine appears promising. Major scientific organizations have suggesting substituting all procedures, when appropriate and feasible, with telemedicine [5].

The hygiene of patients and hospital staff, adequate protective equipment and the investigation, isolation and treatment of COVID-19 patients is the first step to reducing the risk for staff, caregivers and patients.

When there is a suspicion of infection, second level diagnostic procedures are chest imaging with subsequent HRCT, bronchoalveolar lavage (BAL) and COVID-19 test for respiratory viral infections, preferably by RT-PCR.

When diagnosis is made, patients undergo isolation, in hospital if respiratory symptoms are present or in

a domestic setting with strict monitoring if no symptoms are present.

Background in Italy and In Emilia Romagna Region.

At the start of March 2020, the COVID-19 epidemic was announced.

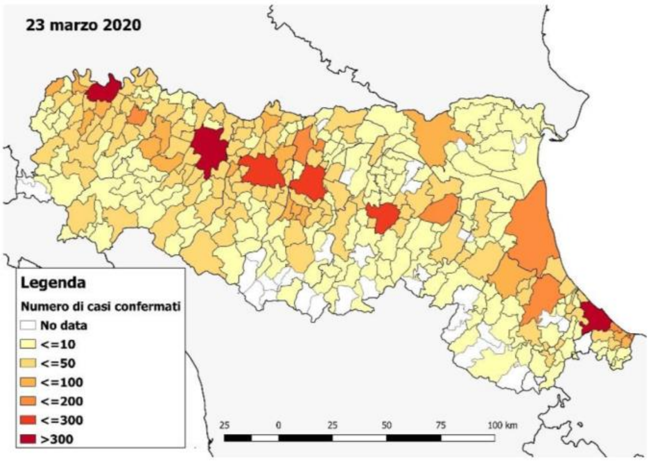

Figure 1. Number of confirmed cases in Emilia Romagna Region (report 23 March 2020)

Since then, the Italian National Institute of Health (Istituto Superiore di Sanità [ISS]) and Emilia Romagna Region have launched a surveillance system to collect information on all people with COVID-19 throughout the country (data reported from all Italian regions/cities, particularly at Emilia Romagna Region, exploring the number of positive tests (via RT-PCR)/deaths with documented positivity/negativity post-previously documented positivity) and has shown, in a subsample of 355 patients with COVID-19 who died in Italy, 87 (24.5%) with active cancer, mainly older patients (no subanalysis about type of malignancy was reported) [6].

Background of IRCCS - IRST Cancer Institute of Romagna (Institutions)

The Cancer Institute of Romagna (IRST- IRCCS) is currently making great breakthroughs in multiple cancer research fields, with outstanding clinical programs, innovative preclinical research, and educational excellence [7]. IRST provides state-of-the-art diagnosis and treatment in a wide range of cancer fields, in line with an excellent healthcare system. The Institute offers precision medicine treatments through a multidisciplinary approach, guaranteeing the most effective interventions for patients.

Established in 2007 in Romagna, IRST serves over 1.1 million inhabitants and ideate, promote and coordinate national and international clinical trials. Fully integrated within the national Public Health System, the institute is a multi-specialty center with high complexity clinical specialties such as radiometabolic therapy, last-generation radiotherapy treatments, cellular therapies, immunotherapy, and a dedicated hospital ward and outpatient day hospital. Within the "Romagna Oncology Network", IRST organizes and steers:

– Oncology research and clinical trials;

– Research infrastructure necessary to promote, conduct and evaluate research with highly skilled dedicated clinicians, researchers, project and grant managers, study coordinators, data-managers and biostatisticians also serving as a CRO for externals;

– Treatments with emerging and innovative technologies;

– Continuous training and development in the field of oncology and hematology.

Background for home care for patients with COVID-19 presenting with mild symptoms and management of their contacts.

WHO has developed this interim guidance to meet the need for recommendations on safe home care for patients with suspected COVID-19 who present with mild symptoms and on public health measures related to the management of their contacts [8].

For those presenting with mild illness, hospitalization may not be possible because of the burden on the health care system, or required unless there is concern about rapid deterioration. If there are patients with only mild illness, providing care at home may be considered, as long as they can be followed up and cared for by family members. Home care may also be considered when inpatient care is unavailable or unsafe (e.g. capacity is limited, and resources are unable to meet the demand for health care services).

In any of these situations, patients with mild symptoms and without underlying chronic conditions − such as lung or heart disease, renal failure, or immunocompromising conditions that place the patient at increased risk of developing complications − may be cared for at home. This decision requires careful clinical judgment and should be informed by an assessment of the safety of the patient's home environment.

In cases in which care is to be provided at home, if and where feasible, a trained Health Care Worker (HCW) should conduct an assessment to verify whether the residential setting is suitable for providing care; the HCW must assess whether the patient and the family are capable of adhering to the precautions that will be recommended as part of home care isolation (e.g., hand hygiene, respiratory hygiene, environmental cleaning, limitations on movement around or from the house) and can address safety concerns (e.g., accidental ingestion of and fire hazards associated with using alcohol-based hand rubs).

If and where feasible, a communication link with health care provider or public health personnel, or both, should be established for the duration of the home care period – that is, until the patient's symptoms have completely resolved.

Patients and household members have been educated about personal hygiene, basic IPC measures, and how to care as safely as possible for the person suspected of having COVID-19 to prevent the infection from spreading to household contacts. The **patient and household members** should be and have been provided with ongoing support and education, and monitoring should continue for the duration of home care. Household members should adhere to the following recommendations.

- Place the patient in a well-ventilated single room (i.e. with open windows and an open door).
- Limit the movement of the patient in the house and minimize shared space. Ensure that shared spaces (e.g. kitchen, bathroom) are well ventilated (keep windows open).
- Household members should stay in a different room or, if that is not possible, maintain a distance of at least 1 metre from the ill person (e.g. sleep in a separate bed).
- Limit the number of caregivers. Ideally, assign one person who is in good health and has no underlying chronic or immunocompromising conditions.
- Visitors should not be allowed until the patient has completely recovered and has no signs or symptoms of COVID-19.
- Perform hand hygiene after any type of contact with patients or their immediate environment.
- Hand hygiene should also be performed before and after preparing food, before eating, after using the toilet, and whenever hands look dirty. If hands are not visibly dirty, an alcohol-based hand rub can be used. For visibly dirty hands, use soap and water.
- When washing hands with soap and water, it is preferable to use disposable paper towels to dry

hands. If these are not available, use clean cloth towels and replace them frequently.

- To contain respiratory secretions, a medical mask should be provided to the patient and worn as much as possible, and changed daily. Individuals who cannot tolerate a medical mask should use rigorous respiratory hygiene; that is, the mouth and nose should be covered with a disposable paper tissue when coughing or sneezing. Materials used to cover the mouth and nose should be discarded or cleaned appropriately after use (e.g. wash handkerchiefs using regular soap or detergent and water).
- Caregivers should wear a medical mask that covers their mouth and nose when in the same room as the patient. Masks should not be touched or handled during use. If the mask gets wet or dirty from secretions, it must be replaced immediately with a new clean, dry mask [8].

Persons (including caregivers and HCWs) who have been exposed to individuals with suspected COVID-19 are considered contacts and should be advised to monitor their health for 14 days from the last day of possible contact.

Definition of a Contact.

A contact is a person who is involved in any of the following from 2 days before and up to 14 days after the onset of symptoms in the patient [9]:

- Having face-to-face contact with a COVID-19 patient within 1 meter and for >15 minutes;
- Providing direct care for patients with COVID-19 without using proper personal protective equipment;
- Staying in the same close environment as a COVID-19 patient (including sharing a workplace, classroom or household or being at the same gathering) for any amount of time;
- Travelling in close proximity with (that is, within 1 m separation from) a COVID-19 patient in any kind of conveyance;
- and other situations, as indicated by local risk assessments.

A way for caregivers to communicate with a healthcare provider should be established for the duration of the observation period. Also, health care personnel should review the health of contacts regularly by phone but, ideally and if feasible, through daily in-person visits, so specific diagnostic tests can be

performed as necessary.

Emergency Access According to National and Emilia Romagna Region procedures. Patients are discouraged to go to Emergency Rooms, if not strictly necessary and are requested to contact there general practitioner.

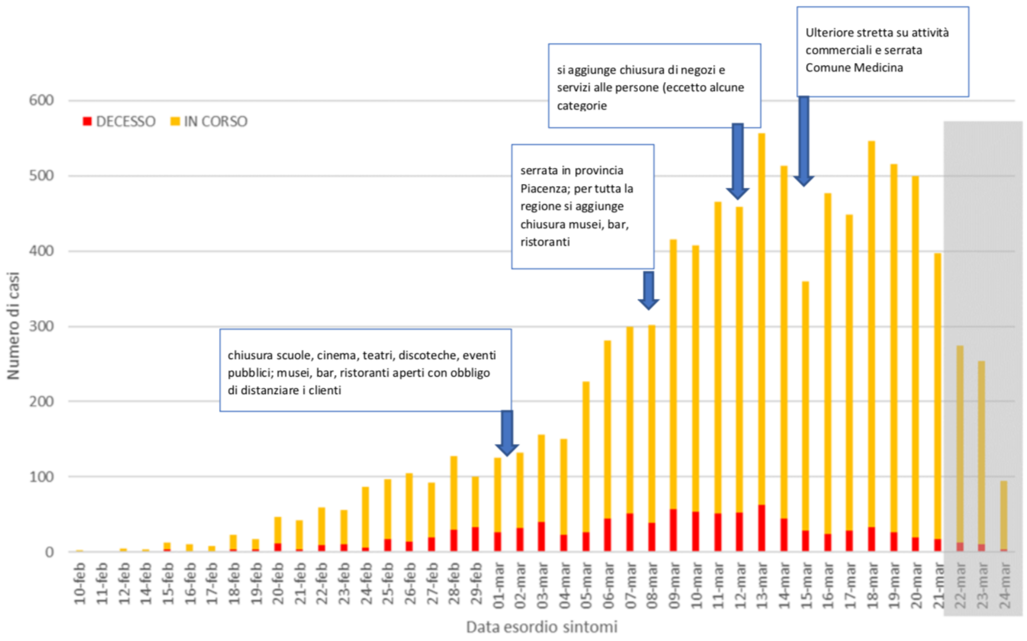

Figure 2. **Number of cases in Emilia Romagna Region (report 24 March 2020)**

Whatever these procedures are entirely applied, (with effect on the infection of COVID-19 on the ER regional as reported on figure) the **necessary of a major containment of COVID-19 infection**, potential pharmacological prophylaxis could be required in very high risk.

1.2 Potential Investigational agent (s) against COVID-19 infection.

Thus, there is an urgent need for an effective treatment to treat asymptomatic patients but also to **decrease the duration of virus carriage** in order to limit the transmission in the community. Among candidate drugs to treat COVID-19, **repositioning of old drugs** for use as antiviral treatment is an interesting strategy because knowledge on safety profile, side effects, posology and drug interactions are well known.

A recent paper reported an inhibitor effect of **remdesivir** [10])(a new antiviral drug) and chloroquine (an old antimalarial drug) on the growth of SARS-CoV-2 *in vitro,* and an early clinical trial conducted in COVID-19 Chinese patients, showed that **chloroquine** [11] had a significant effect, both in terms of clinical outcome and viral clearance, when comparing to controls groups. Chinese experts recommend that patients diagnosed as mild, moderate and severe cases of COVID-19 pneumonia and without contraindications to chloroquine, be treated with 500 mg chloroquine twice a day for ten days [12]. However, there are a number of clinical trials ongoing to study the efficacy of older drugs to be repurposed for use against SARS-CoV-2. One such medication includes the antimalarial chloroquine (CQ), which was recently cited as a potential treatment to shorten SARS-CoV-2 disease course, mitigate inflammatory responses to infection, inhibit the exacerbation of pneumonia, improve lung imaging findings, and promote a virus negative conversion. A number of clinical trials are currently underway as listed on the Chinese Clinical Trial Register to study the efficacy of CQ and its derivatives to treat SARS-CoV-2 (ChiCTR2000029939, ChiCTR2000029935, ChiCTR2000029899, ChiCTR2000029898, ChiCTR2000029868, ChiCTR2000029837, ChiCTR2000029826, ChiCTR2000029803, ChiCTR2000029762, ChiCTR2000029761, ChiCTR2000029760, ChiCTR2000029741, ChiCTR2000029740, ChiCTR2000029609, ChiCTR2000029559, ChiCTR2000029542).

Hydroxychloroquine (an analogue of chloroquine) has been demonstrated to have an anti- SARS-CoV activity *in vitro [13]) and in Vivo [11,14,15]*. There is a long trail of research studies testing the in vitro and in vivo efficacy of chloroquine and its derivatives in treating and preventing infection by various coronavirus species. More recent findings have highlighted the possibility of treating patients infected with the 2019 novel coronavirus, SARS-CoV-2. **Hydroxychloroquine clinical safety profile is better than that of chloroquine** (during long-term use) and allows higher daily dose and has fewer concerns about drug-drug interactions [16,17].

Preclinical Data.

Mechanism of Action of Coronavirus Infection related to the activity of Hydroxychloroquine (HCQ).

Coronaviruses (CoVs) are enveloped, plus-strand RNA viruses belonging to the family Coronaviridae in the order Nidovirales. SARS-CoV-2 has many common features with the coronavirus family and has a phylogenetic similarity to the previous species of SARS-CoV-1 from the outbreak from 2002-2003.

More recent studies found SARS-CoV-2 mimicked SARS-CoV-1 in its severity. Coronavirus populations exhibit considerable genetic heterogeneity, however there are common underlying mechanisms.

Coronavirus delivery of virus particles into the host cell requires binding of the virus to cellular receptors followed by a clathrin-mediated endocytosis to create a viral endosome.

This process is mediated by a **viral surface glycoprotein termed Spike,** a homotrimer of S proteins, binding to the type I integral membrane receptor angiotensin-converting enzyme-2 (ACE2), followed by a pH-independent endocytic reaction (see figure 3 below) [18,19].

It is worth noting that ACE2 is expressed at high levels in type I and II alveolar cells in the lungs.

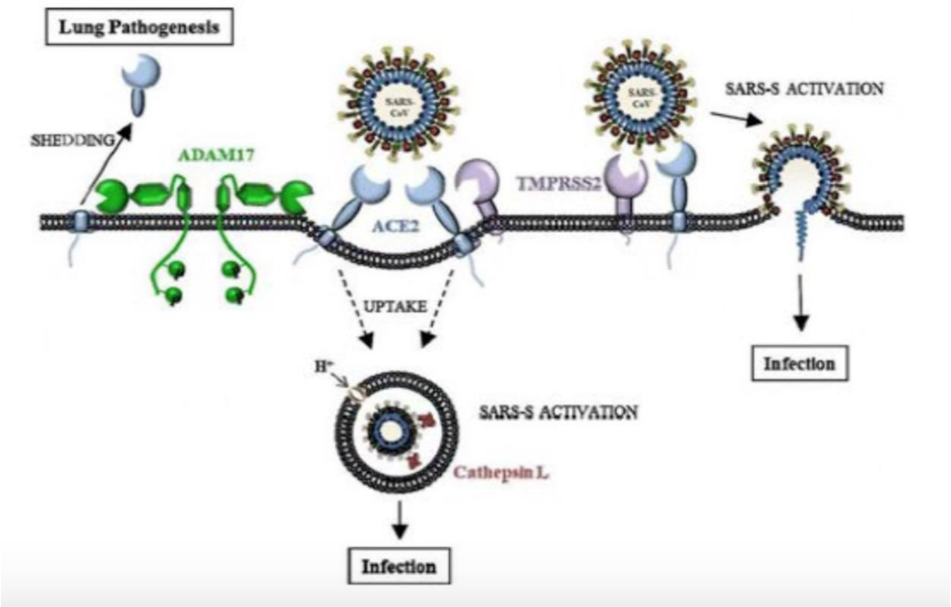

Figure 3 Binding of viral spike protein to receptor ACE-2 [19]

Once internalized, only then does the fusion of virus with lysosomes depend on a **low endosomal and lysosomal pH.**

The S- glycoprotein **is cleaved into S1 and S2 subunits by endosomal proteases cathepsin B and L**, with the resulting S2 subunit mediating membrane fusion.

Cathepsin B and L activity are inhibited by an elevated endosomal pH. Viral entry into the cytoplasm is likewise dependent on an acidic endosomal pH.

Once released into the cytosol, the virus utilizes a **viral RNA-dependent RNA- polymerase (i.e. Replicase)** to drive the viral replication, create virions for exocytosis, and thus further the infection of neighboring cells. **Previous studies** on Human Coronavirus (HCoV- HKU1) indicate that infection of alveolar cells is associated with the surface expression of viral Spike protein, mediating membrane fusion with neighboring cells leading to syncytium formation. This allows direct cell to cell spread of virus which could play a role in the pathogenesis of lung disease and immune system evasion. However, these mechanisms may proceed differently in the novel SARS-CoV-2 as pathogenesis has been shown to differ between coronaviruses [20].

Clinical Outcome of the Infection COVID-19.

Common symptoms of COVID-19 illness include fever, cough, fatigue, muscle pain, dyspnea (i.e. shortness of breath), expectoration, headaches, hemoptysis (blood in the saliva), and diarrhea. Laboratory tests revealed elevated levels of liver enzymes, depleted white blood cell counts, and elevated heart enzymes indicating cardiac impairment. The majority of patients given chest computerized tomography (CT) scans were found to have bilateral ground glass-like opacities and subsegmental areas of consolidation indicative of COVID-19 induced pneumonia. Severe forms of disease are associated with progression to Acute Respiratory Distress Syndrome (ARDS) and septic shock (see figure 4).

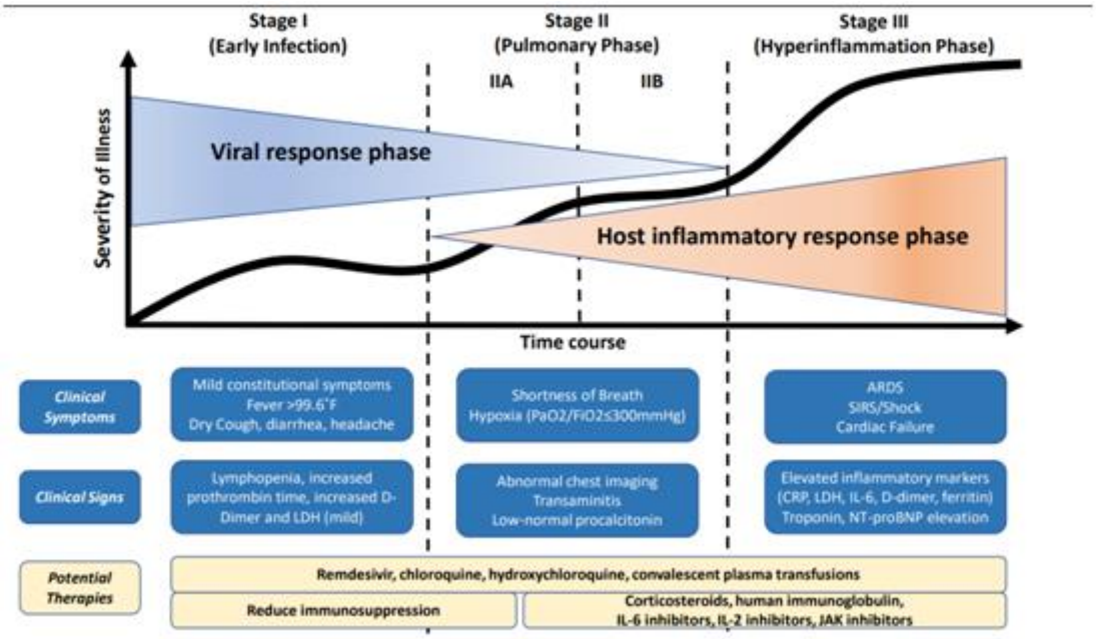

Figure 4 Classification of COVID-19 disease states and potential therapeutic target [21]

In some patients, immune response to the virus resulted in an increase in inflammatory cytokines, which may progress to a “cytokine storm,” followed by multi-organ system dysfunction. For severe cases of disease, more invasive life- saving measures are indicated including admission to the ICU, mechanical ventilation, and extracorporeal membrane oxygenation (ECMO). There is a higher preponderance of severe disease in patients of older age and who have underlying comorbidities such as cardiovascular disease, diabetes, chronic respiratory disease, and oncological diseases [22].

Despite life-saving measures, the case fatality rate (CFR) is calculated to be anywhere between 0.5% and 5.0%, although the actual CFR may differ significantly as the screening and identification of positive cases differs greatly from country to country.

1.3 Investigational agent (s)

Hydroxychloroquine

Several drugs, such as chloroquine have been used in patients with SARS or MERS. Standard assays were carried out to measure the effects of this compound on the cytotoxicity, virus yield and infection rates of 2019-nCoVs. Chloroquine blocked virus infection at low-micromolar concentration and showed high selectivity index.

Chloroquine and Hydroxychloroquine are known to block virus infection by increasing endosomal pH required for virus cell fusion and glycosylation of viral surface proteins. Besides its antiviral activity, chloroquine has an immune-modulating activity, which may synergistically enhance its antiviral effect in vivo [10].

1.4 Clinical Data to Date

Clinical experience on COVID-19 French patients has been recently reported. They planned in a single arm protocol from early March to March 16th, to administer 600mg of hydroxychloroquine daily and the viral load in nasopharyngeal swabs was tested daily in a hospital setting. Presence and absence of virus at Day6-post inclusion was considered the end point. Twenty cases were treated in this study and showed a significant reduction of the viral carriage at D6-post inclusion compared to controls, and much lower average carrying duration than reported of untreated patients in the literature. Azithromycin added to hydroxychloroquine was significantly more efficient for virus elimination [23] (see figure 5)

When comparing the effect of hydroxychloroquine treatment as a single drug and the effect of hydroxychloroquine and azithromycin combination, the proportion of patients that had negative PCR results in nasopharyngeal samples was significantly different between the two groups at days 3-4-5 and 6 post-inclusion. At day6 post-inclusion, 100% of patients treated with hydroxychloroquine and azithromycin combination were virologically cured comparing with 57.1% in patients treated with hydroxychloroquine only, and 12.5% in the control group ($p<0.001$). These results are summarized in Figure 5.

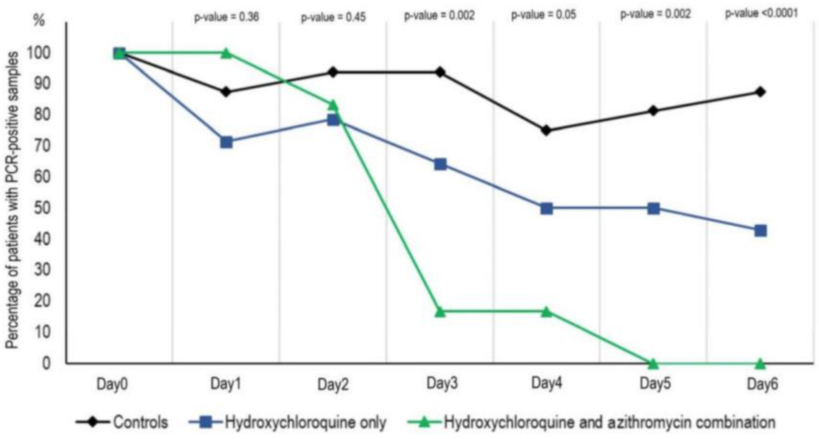

Figure 5. Percentage of patients with PCR-positive nasopharyngeal samples from inclusion to day6 post-inclusion in COVID-19 patients treated with hydroxychloroquine only, in COVID-19 patients treated with hydroxychloroquine and azithromycin combination, and in COVID-19 control patients [23]

Drug effect was significantly higher in patients with symptoms of URTI and LRTI, as compared to asymptomatic patients with $p<0.05$.

1.5 Rationale and Risk/Benefits

In light of these premises, two different populations have been identified which, for different reasons, can be considered candidates to a prospective randomized study aimed at assessing whether the treatment with Hydroxychloroquine reduce the percentage of symptomatic subjects compared to observation only in household members/contacts of COVID-19 patients (Group 1) and if the treatment with Hydroxychloroquine could be introduced in early phase COVID-19 population (Group 2)

2. OBJECTIVES AND OUTCOME MEASURES/ENDPOINTS

2.1 Primary Objectives

Group 1: Prevention of COVID-19 or related symptoms in SARS-CoV-2-exposed subjects, as household members/contacts of COVID-19 patients within one month from randomization

Group 2: Efficacy of Hydroxychloroquine in early phase COVID-19 within 14 days from randomization

2.2 Secondary Objective

1. To compare the efficacy of prophylaxis with Hydroxychloroquine in prevention of COVID-19 infection (swab positive) in a population of SARS-CoV-2-exposed subjects composed by household members/contacts of COVID-19 patients respect to observation only.
2. To assess the efficacy of prophylaxis with Hydroxychloroquine in subgroup population identified by stratification factors, class of age and gender.
3. To assess the efficacy of Hydroxychloroquine in early phase COVID-19 patients within 14 days from randomization in subgroup population identified by stratification factors, class of age and gender
4. To assess the efficacy of Hydroxychloroquine in early phase COVID-19 patients within 1 month from randomization in overall population and in subgroup population identified by stratification factors, class of age and gender.
5. To evaluate treatment toxicity of hydroxychloroquine in SARS-CoV-2-exposed population and COVID-19 patients
6. To evaluate Quality of Life (EQ-5D-5L) from SARS-CoV-2-exposed population and COVID-19 patients
7. To participate in European network for meta-analysis of similar studies or in data collection for identifying prediction rule of outcomes.

2.3 Explorative Objective

To identify biologic features that correlate with susceptibility or resistance to SARS-CoV-2 infection or treatment with Hydroxychloroquine in a subgroup of household members/contacts of COVID-19 patients

or COVID-19 patients enrolled.

2.4 Primary endpoint/outcome

Group 1: The primary endpoint/outcome measure is the proportion of subjects of Group 1 who become symptomatic and/or swab positive in each arm within 1 month from randomization.

Group 2: The primary endpoint/outcome measure is the proportion of subjects of Group 2 who become swab negative in each arm within 14 days from randomization.

2.5 Secondary endpoints/outcomes

1. The proportion of subjects with positive swabs in randomized population of SARS-CoV-2-exposed subjects (Group 1) within 1 month from randomization in both arms.
2. The proportion of subjects of Group 1 who become symptomatic in each arm within 1 month from randomization, in subgroup population identified by stratification factors, class of age and gender.
3. The proportion of subjects of Group 2 who become swab negative in each arm within within 14 days from randomization, in subgroup population identified by stratification factors, class of age and gender.
4. The proportion of subjects of Group 2 who become swab negative in each arm within 1 month from randomization in overall population and in subgroup population identified by stratification factors, class of age and gender.
5. Absolute and relative frequencies of Serious Adverse Events (CTCAE version 5.0) in both arms for the Group 1 and Group 2.
6. Variation in Quality of Life scores in different time points (weekly) respect to baseline values in both Group 1 and Group 2 populations.

3. STUDY DESIGN

3.1 Summary of Trial Design

This is an open label, superiority, cluster-randomized Italian interventional clinical trial, evaluating the role of Hydroxychloroquine versus observation only in preventing infection to COVID-19 or treating early phase COVID-19 patients.

The Cluster-randomization, around index case, is considered the most appropriate design, as the lack of independence among participants cannot be excluded. In this study the index case is a person newly diagnosed with COVID-19 and relative cluster is composed by SARS-CoV-2-exposed subjects, as household members and contacts of the COVID-19 patient

Each index case is randomised to either Arm A: Hydroxychloroquine or Arm B: observation in a 2:1 ratio on an open label basis. Participants in the same cluster receive the same intervention.

All participating subjects becoming paucisymptomatic/symptomatic can continue/start, out of random, hydroxychloroquine, if not otherwise treated by treating physician or if not contraindicated.

Study population is constituted by:

Group 1: SARS-CoV-2-exposed subjects, as household members/contacts of COVID-19 patients.

Group 2: Patients with COVID-19 asymptomatic or paucisymptomatic in home situation.

For Group 1:

A sample size of about 2000 SARS-CoV-2-exposed subjects, as household members/contacts of COVID-19 patients will participate into the study. Assuming about 1.5-2.0 asymptomatic household members/contacts for each COVID-19 patient, we expected to identify approximately 1000-1300 COVID-19 index cases.

For Group 2:

Sufficient power for primary objective (negative swab within 14 days from randomization) will be reached, given a sample size of 300 COVID-19 subjects asymptomatic or paucisymptomatic in home situation not treated for COVID-19 (25-30% of about 1000-1300 expected case index COVID-19 patients).

Since up to date reduced evidences about COVID-19 infection epidemiology, the continuous update of diagnostic and therapeutic approaches, the sample size estimation could be updated after a one third of the population will be recruited and eventually modified according a substantial protocol amendment.
The overall study duration will be 10 months; 3 months for subjects enrollment, and 1 month of treatment and further 6 months of follow-up.

3.2 End of trial definition

The end of trial will be the date after the last data capture. The Promoter will notify the IEC(s) that the trial has ended and a summary of the clinical trial report will be provided within 12 months of the end of trial.

4. STUDY POPULATION

Group 1:

SARS-CoV-2-exposed subjects, as household members and/or contacts of COVID-19 patients.

Group 2:

Patients with COVID-19 asymptomatic or paucisymptomatic in home situation

Trial subjects must meet all inclusion and exclusion criteria. Subjects eligibility will be checked before randomization by the trial personnel as described in detail in section 5 (Study Procedures).
In addition, the subject must be thoroughly informed about all aspects of the study, including the study visit schedule and required evaluations and all regulatory requirements for informed consent. The informed consent must be obtained from the subject prior to enrollment. The following criteria apply to all subjects enrolled onto the study unless otherwise specified.

4.1 Inclusion Criteria

1. Male or Female, aged >= 18 years
2. SARS-CoV-2-exposed subjects, as household members and/or contacts of COVID-19 patients (Group 1). In this group are included Health care professionals in contact with COVID-19 patients.

or

3. COVID-19 patients, asymptomatic or paucisymptomatic in home situation who are not in treatment

with any anti COVID-19 medication (Group 2)

4. Absence of any COVID-19 symptom in last week before randomization (fever >37.5°C, cough, dyspnea) (only for group 1 subjects)
5. Paracetamol treatment is accepted only for group 2.
6. Participant is willing and able to give informed consent for participation in the study (either recorded during a telephonic interview or signed in person) and agrees with the study and its conduct.

4.2 Exclusion Criteria

The participant may not enter the study if ANY of the following apply:

1. Reported anamnesis for:
   a. Intolerance or previous toxicity for hydroxychloroquine/chloroquine
   b. Bradycardia or reduction rhythm of heart with arrythmias
   c. Ischemic heart disease
   d. Retinopathy
   e. Congestive heart failure under/with use of diuretics
   f. Favism or glucose-6-phosphate dehydrogenase (G6PD) deficiency
   g. Diabetes type 1
   h. Major comorbidities like advanced chronic kidney disease or dialysis therapy, known history of ventricular arrhythmias, any oncologic/hematologic malignancy.
   i. Severe neurological and mental illness
2. Any other contraindication to take hydroxychloroquine
3. Already taking chloroquine, hydroxychloroquine or analogous during the past 3 weeks
4. Use of other antiviral agents in the last 3 weeks
5. Known positiveness for HIV, active HCV, HBV infection
6. Subject with a positive test for SARS-CoV-2 (for Group 1)
7. Pregnant or lactating
8. Current use of medications with known significant drug-drug interactions: digoxin, hypoglycemic agents, anticonvulsant, Cyclosporine, Phenylbutazone, drugs that inhibit CYP2D6
9. Known prolonged QT syndrome or current use of drugs with known QT prolongation

10. Participation in another clinical trial with any investigational agents within 30 days prior to study screening.

5. STUDY PROCEDURES

See for schedule of procedures Appendix B.

5.1 Identification of eligible subjects

The Public Hygiene, Infectious Diseases Units and General Practitioners are in charge of contacting all subjects who have come into contact with patients diagnosed with COVID-19 within a period of 48 hours before the onset of COVID-19 symptoms. These subjects are contacted by telephone and are asked to quarantine and to report any symptom. Furthermore, all healthcare professionals who have had contacts with COVID-19 patients will be considered.

Any asymptomatic subject cohabitant with COVID-19 patient and any asymptomatic or pauci symptomatic patient with COVID-19 not treated with any specific medication, will be contacted by the Public Hygiene collaborator and/or the general practitioner and/or Infectious Disease specialists , and will be asked if he/she is interested in participating to the PROTECT clinical trial. The Public Hygiene collaborator and/or the general practitioner and/or Infectious Disease specialists will provide to the coordinating center IRST IRCCS, on a daily basis, a list of potentially eligible subjects who agree to be re-contacted with corresponding contact details.

The Trial Personnel will contact all eligible subjects by telephone, will give detailed information about the study, will check for subject eligibility and will record informed consent to participate.

A webpage with informative material will be available at www.irst.emr.it

5.2 Eligibility check

All inclusion and exclusion criteria will be checked before subject inclusion by the trial personnel as described during telephonic interviews with the subject him/herself. An accurate anamnestic evaluation will be performed. Specific questionnaires regarding cardiac risk factors, history of any allergy, other relevant clinical conditions will be administered to subjects to evaluate any contraindication to the use of Hydroxychloroquine. When necessary, the general practitioner will be contacted for further information. Furthermore, additional information will be retrieved by administrative databases of Hospitalizations

(SDO-Schede di Dimissione Ospedaliera), outpatient Specialistic Procedures (ASA-Assistenza Specialistica Ambulatoriale), drugs (FED-Farmaci ad Erogazione Diretta and AFT-Assistenza Farmaceutica Territoriale) that patient could have received starting from 3 years before the enrollment. Research for administrative information crossed with information given orally and through the questionnaires by the patient, will allow to reconstruct the medical history of the participant to the trial.

5.3 Informed Consent

According to Good Clinical Practice and to European and Italian laws concerning clinical trials, all subjects must personally give written informed consent to participate to a clinical trial before any study procedure. However, given the extraordinary nature of moment due to pandemic diffusion of COVID-19 in Italy and the lockdown imposed by the DPCM 11.03.2020, it is not feasible to provide information to the potentially eligible subjects through a face-to-face interview and to collect a signed informed consent by the patient. As an alternative to written consent, Reg.EU 536, (art 29) allows to record the informed consent through appropriate alternative means when the subject is unable to write. Furthermore, art. 35 “Clinical trials in emergency situations” states that in cases when “due to the urgency of the situation, caused by a sudden life-threatening or other sudden serious medical condition, the subject is unable to provide prior informed consent” it is possible to collect informed consent after the inclusion of patient in the trial.

Taking into account all these considerations, the following procedure to collect informed consent will be applied in this trial:

- study brochure, detailed participant information sheet and other explicative materials (including but not limited to videos, powerpoint presentations etc) will be available on the IRST IRCCS website (www.irst.emr.it), in the study web page, that will be easily retrievable by the homepage. All the materials must receive approval by the competent Ethic Committee.

All these electronic informative materials will contain detailed information on: the exact nature of the study; the implications and constraints of the protocol; the known side effects and any risks involved in taking part, in a language that will be easily understood by a lay person. It will be clearly stated that the participant is free to withdraw from the study at any time for any reason without prejudice to future care, and with no obligation to give the reason for withdrawal.

At first telephonic contact all potentially eligible subjects will be invited to visit the study webpage.

The Informed Consent process will be conducted by means of recording telephonic interview with the subject. A medical doctor of the trial personnel/study team, suitably qualified and experienced, and authorised by the Chief/Principal Investigator, will call the potentially eligible subjects who have expressed interest to participate, and will explain the study, will give them all the information and answer to all the subjects' questions. After this telephonic interview, if the subject confirms his/her wish to participate, the IRST medical doctor will record the patient's consent with appropriate instruments. To do so, the medical doctor will first of all ask the subjects to confirm their identity by clearly pronouncing their generalities; then the subjects will be asked to answer some questions regarding: comprehension of the study treatment, procedures and risk/benefit; agreement to participate to the study. The patient will give his/her consent also to access to all administrative information (on procedures, activities, hospitalizations, use of drugs) that are collected with administrative scope in regional database. The interview will be recorded. All subjects must be informed and must consent to the recording of their interview before proceeding. The medical doctor conducting the interview will complete a form with the subject's answers. The medical doctor will then print, sign and date the form. A copy of the form signed by the medical doctor who conducted the telephonic interview will be sent by mail to the participants. The original signed form will be retained at IRST IRCCS. The participants will be allowed as much time as wished to consider the information, and the opportunity to question the Investigator, or other independent parties to decide whether they will participate in the study. In case they ask for more time, they will be re-contacted to record the consent in a separate moment.

Whenever possible a signed informed consent sheet will be obtained by the subjects (e.g. in the case the participant will be consulted to the doctor personally).

5.3 Registration and Assignment of Subject ID

The person who obtained the consent will assign an Identification code to the subject (alphanumeric ID). The subject ID will be sent by email to the subject and recorded in the patient log conserved at IRST IRCCS. The Coordinating center staff will register the subject ID in the study electronic Case report form (CRF). Only the subject ID will be used to register clinical information on eCRF.

5.4 Screening and Eligibility Assessments

The Screening procedures and assessments will be completed through telephonic interview with participants and, if required with the general practitioners and/or administrative databases search.

A template for the telephonic interviews will be prepared, containing all the issues to be addressed.

The following information will be recorded for all subjects:

- Demographics, year of birth, gender, ethnic origin, city of residence
- Complete Medical history, including comorbidities and concomitant medications
- general health state
- Body temperature
- Presence of any symptom compatible with COVID-19 in the 15 days before enrollment (fever, coughing, sore throat and shortness of breath, disgeusia, diarrea, vomit);
- Quality of Life questionnaire (EQ-5D-5L);
- If available, results from the most recent blood examinations (blood count, glucose level, PCR, liver and kidney functionality, coagulation panel)
- Details about exposure to COVID-19 patients, including type and frequency of contact, travelling data in the last 15 days before enrollment (Group 1);
- Date of rhino/oro-pharyngeal swab, if performed

5.5 Randomisation

All Households members and/or contacts fulfilling all inclusion criteria (for Group 1) of each COVID-19 patient, will be enumerated into a single cluster (information of each subject will be recorded in specific data record) and these clusters will be cluster-randomised (2:1) to either arm A or arm B.

Randomization lists will be stratified according to the following factors:

- COVID-19 risk level for residence (high vs low/intermediate);
- Health care professionals (yes vs no)
- Home situation without COVID-19 treatment (yes vs no)

COVID-19 index cases will be randomized (2:1) to either arm A or arm B.

An independent statistician not otherwise involved in the trial will generate the allocation sequence, and COVID-19 response teams will be unaware of the allocation of clusters.

5.6 Assessments during treatment/observation

Group 1 randomized subjects will record the following information on a daily diary and will be monitored with telephonic interviews every week for the duration of treatment/observation (one month).

Group 2 randomized patients will record the following information on a daily diary and will be monitored with telephonic interview every other day for the first week and then every week for up to one month.

During the interview the following information will be asked and recorded: :

- compliance with study drug (only for subjects randomized to receive hydroxychloroquine);
- changes in body temperature in the last 7 days;
- general health state;
- onset (for Group 1) or change (for Group 2) of any symptom compatible with COVID-19 (fever, coughing, sore throat and shortness of breath, dysgeusia, diarrhea, vomit);
- in case rhino/oro-pharyngeal swab has been performed (group 1), date and result of the test; for subjects of Group 2, rhino/oro-pharyngeal swab will be performed within 14 days from study entry and then after 30 days;
- any adverse event occurred in the last seven days or any update regarding an adverse event occurred from study start;
- any contact with COVID-19 patient (other than the cohabitant for group 1) or any risk situation occurred in the last 7 days;
- Quality of life Questionnaires (EQ-5D-5L) (every week);
- concomitant medications.

5.7 Follow-up assessments

Participants will be followed every 4 weeks for up to 6 months after one month from randomization. Telephonic interviews will be performed, asking information on:

- changes in body temperature in the last 4 weeks;
- general health state (performance status will be desumed by information provided by the subject);
- onset (Group 1) or change (Group 2) of any symptom compatible with COVID-19 (fever, coughing, sore throat and shortness of breath, dysgeusia, diarrhea, vomit);
- in case rhino/oro-pharyngeal swab has been performed, date and result of the test;
- any adverse event occurred within one month after treatment stop;
- any contact with COVID-19 patient (other than the cohabitant for group 1) or any risk situation

occurred in the past 4 weeks;

- Quality of life Questionnaires (EQ-5D-5L);
- recording of concomitant medications.

5.8 Discontinuation/ Withdrawal of Participants from Study Treatment

Each participant has the right to withdraw study at any time. In addition, the investigator may discontinue a participant from the study at any time if the investigator considers it necessary for any reason including:

All subjects becoming paucisymptomatic/symptomatic, can continue/start, out of random, hydroxychloroquine,if not otherwise treated by treating physician or if not contraindicated.

- Subjects of Group 1 resulting in COVID-19 Positive, during or after study period, who will be start treatment according to National and regional guidelines, will be followed - if he/she consent- with registration of biological and clinical data of outcome for the next 3 and 6 months or until withdrawal of informed consent.
- Subjects of Group 2 who become symptomatic or have an increased worsening of symptoms, require a change in treatment within 14 days of randomisation, are failures for primary and secondary objectives.
- Ineligibility (either arising during the study or retrospective having been overlooked at screening)
- General or specific changes in the subject's condition render the patient unacceptable for further treatment in the judgment of the investigator.
- Significant protocol deviation
- Significant non-compliance with treatment regimen or study requirements
- An adverse event which requires discontinuation of the study medication or results in inability to continue to comply with study procedures
- Pregnancy
- Consent withdrawn
- Lost to follow up

The reason for withdrawal will be recorded in the CRF.

If the participant is withdrawn due to an adverse event, the investigator will arrange for follow-up visits or telephone calls until the adverse event has resolved or stabilised.

5.9 Source Data

Source documents are original documents, data, and records from which participants' CRF data are obtained. These include, but are not limited to, patients diaries, hospital records (from which medical history and previous and concurrent medication may be summarised into the CRF), clinical and office charts, laboratory and pharmacy records, microfiches, radiographs, and correspondence.

CRF entries will be considered source data if the CRF is the site of the original recording (e.g., there is no other written or electronic record of data). In this study the CRF will be used as the source document for medical history, comorbidities, informations regarding exposure to SARS-CoV-2, and all the informations collected through telephonic interviews.

All documents will be stored safely in confidential conditions. On all study-specific documents, other than the signed consent, the participant will be referred to by the study participant number/code, not by name.

Direct access will be granted to authorised representatives from the sponsor/promoter, host institution and the regulatory authorities to permit trial-related monitoring, audits and regulatory inspections, including provision of direct access to source data and documents.

Other source data will include the administrative databases referred to health care activities received by the patient and recorded in the following databases: Hospitalizations (SDO-Schede di Dimissione Ospedaliera), outpatient Specialistic Procedures (ASA-Assistenza Specialistica Ambulatoriale), drugs (FED-Farmaci ad Erogazione Diretta and AFT-Assistenza Farmaceutica Territoriale)Access to these databases will be allowed by the Regional Health Care authorities, that will provide the unique numeric identification codes of the enrolled patients (Prog_paz). Through a complex procedure of data linkage, data on each Prog_paz key will be searched and retrieved in each database.

6. STUDY TREATMENT

Hydroxychloroquine (HCQ) sulfate, a derivative of CQ, was first synthesized in 1946 by introducing a hydroxyl group into CQ and was demonstrated to be much less (~40%) toxic than CQ in animals [13].

Hydroxychloroquine, (hydroxychloroquine sulfate tablets) is indicated for the treatment of rheumatoid arthritis, and discoid and systemic lupus erythematosus, in patients who have not responded satisfactorily to drugs with less potential for serious side effects.

It is also indicated for the suppressive treatment and treatment of acute attacks of malaria due to P. vivax,

P. malariae, P. ovale, and susceptible strains of P. falciparum. It is not active against the exo-erythrocytic forms of P. vivax, P. malariae and P. ovale and therefore will neither prevent infection due to these organisms when given prophylactically, nor prevent relapse of infection due to these organisms. It is highly effective as a suppressive agent in patients with vivax or malariae malaria in terminating acute attacks and significantly lengthening the interval between treatment and relapse. In patients with falciparum malaria, it abolishes the acute attack and effects complete cure of the infection, unless due to a resistant strain of P. falciparum.

Hydroxychloroquine has been demonstrated to have an anti- SARS-CoV activity in vitro [13] and in Vivo. There is a long trail of research studies testing the in vitro and in vivo efficacy of chloroquine and its derivatives in treating and preventing infection by various coronavirus species. More recent findings have highlighted the possibility of treating patients infected with the 2019 novel coronavirus, SARS-CoV-2.

The planned treatment schedules are based on the following criteria:

Group 1: Antimalarial prophylaxis) according to [24]

Group 2: Region Emilia Romagna recommendation ("Protocollo terapeutico per la terapia antivirale dei pazienti con infezione da COVID-19").

6.1 Description of Study Treatment

Arm A) Hydroxychloroquine

Group1:

A loading dose Hydroxychloroquine 400 mg twice daily at day 1, followed by a weekly dose of Hydroxychloroquine 200 mg twice daily on days 8, 15 and 22, covering a total of 1 month of treatment.

Group 2:

A loading dose Hydroxychloroquine 400 mg twice daily at day 1 followed by 200 mg twice daily for a total of at least 5-7 days according to clinical evolution.

6.2 Supply of study treatment

<u>Hydroxychloroquine</u>

Product description:

Hydroxychloroquine will be provided as white to off-white, film coated, peanut-shaped tablets, containing 200 mg hydroxychloroquine sulfate (equivalent to 155 mg base)

Storage requirements:

Hydroxychloroquine must be stored at room temperature (15°C -30°C).

Route of administration:

Hydroxychloroquine should be taken every 12 hours with food or milk.

Expected adverse events:

The most common adverse of the drug are gastrointestinal effects, including nausea, vomiting, diarrhea and abdominal discomfort. An important consideration is that several studies have reported the incidence of cardiotoxic effects, including rhythm disorders (such as a prolonged QT interval) and the development of cardiomyopathy in patients with rheumatic diseases, but conclusive evidence is lacking and further pharmacovigilance is required.

The most severe complication attributed to antimalarial treatment is the development of retinopathy with prolonged use as these drugs can cause retinal damage by disrupting an important step in the visual cycle mediated by lysosomal degradation. Retinopathy is more commonly associated with CQ than with HCQ and can result in patients developing retinal defects including circular and diametric defects.

6.3 Compliance with Study Treatment (if applicable)

The experimental drug will be sent together with a drug diary to every subject enrolled in the protocol

6.4 Accountability of the Study Treatment

The study drug will be supplied by IRST IRCCS Pharmacy to each participant of the study. All movements of study medication from IRST IRCCS pharmacy will be documented.

The experimental drug will be delivered to the home of the study participants with a delivery document which must be signed by the subject for receipt.

6.5 Concomitant Medication

During the first contact with the subject, PI carries out the pharmacological survey and transfers the drug's list of the patient in CRF.

Throughout the study Investigators may prescribe any concomitant medications or treatments deemed necessary to provide adequate supportive care. Any medication, other than the study medication taken during the study will be recorded in the CRF.

Prohibited concomitant medications (htts://www.covid19-druginteraction.org/)

**Antiarrhythmics**	**Antimicrobics**	**Antidepressants**	**Antipsycho tic**	**Analgesics**	**Anticonvulsants**
Amiodarone	Rifampicin	St John's wort	Ziprasidone	Dextropropoxyphene	Carbamazepine
Flecainide	Rifapentine			Metamizole	Phenobarbital
Bepridil					Phenytoin
Mexiletine					Primidone

If drug is not listed it cannot automatically be assumed it is safe to co- administer. Drug interaction data for many agents are limited or absent; therefore, risk/benefit assessment for any individual patient rests with prescribers.

7. DOSING DELAYS / DOSE MODIFICATIONS

Not applicable

7.1 Study treatment modifications

No dose modification is foreseen.

Toxicity will be assessed according to the NCI Common Terminology Criteria for Adverse Events Version 5.0 (CTCAE v5.0)

8. BIOMARKER / CORRELATIVE / SPECIAL STUDIES

A peripheral blood specimen (7 cc) will be collected from a subset of subjects enrolled and stored to perform future biological analysis to identify potential factors correlated with susceptibility/resistance to infection and with sensitivity and resistance to Hydroxychloroquine. Samples may be also used to test new serological assays - should they become available - to identify virus-specific antibodies.

During the informed consent process, each subject will be asked if he/she agrees to donate a blood sample for future studies regarding biological factors underlying infection by SARS-CoV-2. Whenever it will be possible to perform blood sampling in safe conditions, subjects will be contacted for sampling. As an alternative, if it will be possible, blood sampling will be performed at patient's home whenever the trial personnel will go to perform rhino/oro-pharyngeal swabs. A specific informed consent for this biological substudy will be collected from subjects before the sample collection. Samples will be stored at IRST Bioscience laboratory.

9. SAFETY REPORTING

Analyses will be performed for all patients having received at least one dose of study drug. The National Cancer Institute's Common Terminology Criteria for Adverse Events (CTCAE) Version 5.0 should be used to assess and grade AE severity, including laboratory abnormalities judged to be clinically significant. If the experience is not covered in the modified criteria, the guidelines shown in the table below should be used to grade severity. It should be pointed out that the term "severe" is a measure of intensity and that a severe AE is not necessarily serious.

AE Severity Grading

Severity (Toxicity Grade)	Description
**Mild (1)**	Transient or mild discomfort; no limitation in activity; no medical intervention or therapy required. The subject may be aware of the sign or symptom but tolerates it reasonably well.
**Moderate (2)**	Mild to moderate limitation in activity, no or minimal medical intervention/therapy required.
**Severe (3)**	Marked limitation in activity, medical intervention/therapy required, hospitalizations possible.
**Life-threatening (4)**	The subject is at risk of death due to the adverse experience as it occurred. This does not refer to an experience that hypothetically might have caused death if it were more severe.

9.1 Definitions

Adverse Event (AE): Any untoward medical occurrence in a patient or clinical investigation participants administered a medicinal product, which does not necessarily have to have a causal relationship with this treatment (the study medication).

An AE can therefore be any unfavourable and unintended sign (including an abnormal laboratory finding), symptom or disease temporally associated with the use of the study medication, whether or not considered related to the study medication.

Adverse Reaction (AR): All untoward and unintended responses to a medicinal product related to any dose.

The phrase "responses to a medicinal products" means that a causal relationship between a study medication and an AE is at least a reasonable possibility, i.e., the relationship cannot be ruled out.

All cases judged by either the reporting medically qualified professional or the sponsor as having a reasonable suspected causal relationship to the study medication qualify as adverse reactions.

Severe Adverse Events: To ensure no confusion or misunderstanding of the difference between the terms "serious" and "severe", which are not synonymous, the following note of clarification is provided:

The term "severe" is often used to describe the intensity (severity) of a specific event (as in mild, moderate, or severe myocardial infarction); the event itself, however, may be of relatively minor medical significance (such as severe headache). This is not the same as "serious," which is based on patient/event outcome or action criteria usually associated with events that pose a threat to a participant's life or functioning. Seriousness (not severity) serves as a guide for defining regulatory reporting obligations.

Serious Adverse Event (SAE) or Serious Adverse Reaction: A serious adverse event or reaction is any untoward medical occurrence that at any dose:

- Results in death,
- Is life-threatening*,
- Requires inpatient hospitalisation or prolongation of existing hospitalisation**,
- Results in persistent or significant disability/incapacity, or
- Is a congenital anomaly/birth defect.
- Or is otherwise considered medically significant by the Investigator***

Comments:

The term severe is often used to describe the intensity (severity) of a specific event. This is not the same as serious, which is based on patients/event outcome or action criteria.

* Life threatening in the definition of an SAE refers to an event in which the patient was at risk of death at the time of the event; it does not refer to an event that hypothetically might have caused death if it were more severe.

**Hospitalisation Is defined as an unplanned, formal inpatient admission, even if the hospitalisation is a precautionary measure for continued observation. Thus, hospitalisation for protocol treatment (e.g. line insertion), elective procedures (unless brought forward because of worsening symptoms) or for social reasons (e.g. respite care) are not regarded as an SAE.

*** Medical judgment should be exercised in deciding whether an AE is serious in other situations. Important AEs that are not immediately life threatening or do not result in death or hospitalisation but may jeopardise the subject or may require intervention to prevent one of the other outcomes listed in the definition above, should be considered serious.

Suspected Unexpected Serious Adverse Reactions (SUSAR): A serious adverse reaction, the nature or severity of which is not consistent with the applicable product information (e.g., Investigator's Brochure for an unapproved investigational product or package insert/summary of product characteristics for an approved product).

Expected Serious Adverse Events/Reactions: The most common adverse of the drug are gastrointestinal effects, including nausea, vomiting, diarrhea and abdominal discomfort. An important consideration is that several studies have reported the incidence of cardiotoxic effects, including rhythm disorders (such as a prolonged QT interval) and the development of cardiomyopathy in patients with rheumatic diseases, but conclusive evidence is lacking and further pharmacovigilance is required.

The most severe complication attributed to antimalarial treatment is the development of retinopathy with prolonged use as these drugs can cause retinal damage by disrupting an important step in the visual cycle mediated by lysosomal degradation. Retinopathy is more commonly associated with CQ than with HCQ and can result in patients developing retinal defects including circular and diametric defects.

Adverse Event Reporting Period

The study period during which adverse events must be reported is normally defined as the period from the initiation of any study procedures to the end of the study treatment follow-up. For this study, the study treatment follow-up is defined as 30 days following the last administration of study treatment.

Pre-Existing Condition

A preexisting condition is one that is present at the start of the study. A preexisting condition should be recorded as an adverse event if the frequency, intensity, or the character of the condition worsens during the study period.

Post-study Adverse Event

All unresolved adverse events should be followed by the investigator until the events are resolved, the subject is lost to follow-up, or the adverse event is otherwise explained. At the last scheduled visit, the investigator should instruct each subject to report any subsequent event(s) that the subject, or the subject's personal physician, believes might reasonably be related to participation in this study. The investigator should notify the study sponsor/promoter of any death or adverse event occurring at any time after a subject has discontinued or terminated study participation that may reasonably be related to this study. The sponsor/promoter should also be notified if the investigator should become aware of the development of cancer or of a congenital anomaly in a subsequently conceived offspring of a subject that has participated in this study.

Hospitalization, Prolonged Hospitalization or Surgery

Any adverse event that results in hospitalization or prolonged hospitalization should be documented and reported as a serious adverse event unless specifically instructed otherwise in this protocol.

The hospitalization for worsening of the clinical condition or the symptoms related to COVID-19 should be documented and reported as a serious adverse event.

Any condition responsible for surgery should be documented as an adverse event if the condition meets the criteria for and adverse event.

Neither the condition, hospitalization, prolonged hospitalization, nor surgery are reported as an adverse event in the following circumstances:

- Hospitalization or prolonged hospitalization for diagnostic or elective surgical procedures for a preexisting condition. Surgery should ***not*** be reported as an outcome of an adverse event if the purpose of the surgery was elective or diagnostic and the outcome was uneventful.
- Hospitalization or prolonged hospitalization required to allow efficacy measurement for the study.
- Hospitalization or prolonged hospitalization for therapy of the target disease of the study, unless it is a worsening or increase in frequency of hospital admissions as judged by the clinical investigator

9.2 Reporting Procedures for All Adverse Events

All AEs occurring during the study reported by the participant, whether or not attributed to study medication, will be recorded on the CRF starting from the date informed consent is recorded.

The following information will be recorded: description, date of onset and end date, severity (according to CTCAE version 5.0), assessment of relatedness to study medication, other suspect drug or device and action taken. Follow-up information should be provided as necessary.

AEs considered related to the study medication as judged by a medically qualified investigator or the sponsor/promoter will be followed until resolution or the event is considered stable. All related AEs that result in a participant's withdrawal from the study or are present at the end of the study, should be followed up until a satisfactory resolution occurs.

Serious adverse events that are still ongoing at the end of the study period must be followed up to determine the final outcome. Any serious adverse event that occurs after the study period and is considered to be possibly related to the study treatment or study participation should be recorded and reported immediately.

It will be left to the investigator's clinical judgment whether or not an AE is of sufficient severity to require the participant's removal from treatment. A participant may also voluntarily withdraw from treatment due to what he or she perceives as an intolerable AE. If either of these occurs, the participant must undergo an end of study assessment and be given appropriate care under medical supervision until symptoms cease or the condition becomes stable.

The relationship of AEs to the study medication will be assessed by a medically qualified investigator.

Relationship to Drug	Comment
Definitely	Previously known toxicity of agent; or an event that follows a reasonable temporal sequence from administration of the drug; that follows a known or expected response pattern to the suspected drug; that is confirmed by stopping or reducing the dosage of the drug; and that is not explained by any other reasonable hypothesis.
Probably	An event that follows a reasonable temporal sequence from administration of the drug; that follows a known or expected response pattern to the suspected drug; that is confirmed by stopping or reducing the dosage of the drug; and that is unlikely to be explained by the known characteristics of the subject's clinical state or by other interventions.

Possibly	An event that follows a reasonable temporal sequence from administration of the drug; that follows a known or expected response pattern to that suspected drug; but that could readily have been produced by a number of other factors.
Unrelated	An event that can be determined with certainty to have no relationship to the study drug.

AE Relationship to Study Drug

Any pregnancy occurring during the clinical study and the outcome of the pregnancy, should be recorded and followed up for congenital abnormality or birth defect.

9.3 Reporting Procedures for Serious Adverse Events

The Investigator is responsible for reporting all Serious Adverse Events (SAE), related or not to the study treatment, occurring during from the date the patient signs the informed consent to 30 days after the last protocol treatment, to the "Safety Desk". Any late Serious Adverse Drug Reaction (SADR), occurring after this 30-day period, should follow the same reporting procedure.

If a SAE occurs, the following action must be taken by the investigator:

Fill in the SAE form and send by fax within 24 hours of the initial observation of the event, to the sponsor/promoter:

IRST Safety Desk
FAX 0543 739288
e-mail: fv.ct@irst.emr.it

- Attach a report of the event and a copy of all examinations that were carried out, including the dates on which these examinations were performed. For laboratory tests, normal laboratory ranges must also be included.
- All forms must be dated and signed by the responsible investigator or one of his/her authorized staff members.
- Additional information received for a case (follow-up or corrections to the original case) need to be detailed on a new SAE form and faxed to IRST.
- IRST Safety Desk will perform an initial check of the information and ensure that it is reviewed by the responsible safety physician.

- The IRST safety desk will send the SAE report to national authorities, Ethical Committees and investigators as appropriate, according to local regulations.
- IRST will report all SUSARs to the Competent Authorities and the Ethical Committees concerned. Fatal or life-threatening SUSARs must be reported within 7 days and all other SUSARs within 15 days. IRST will also inform all investigators concerned of relevant information about SUSARs that could adversely affect the safety of participants.
- In addition to the expedited reporting above, the IRST Safety Desk shall submit once a year throughout the clinical trial or on request a safety report to the Competent Authority and Ethical Committees.

10. STATISTICAL CONSIDERATIONS

Prior to the analysis of the final study data, a detailed Statistical Analysis Plan (SAP) will be written describing all analyses that will be performed. The SAP will contain any modifications to the analysis plan described below.

10.1 Sample Size, Accrual Rate and Study Duration

Group 1: The primary endpoint/outcome measure is the proportion of subjects of Group 1, who become symptomatic and/or swab positive (binary outcome) in each arm within 1 month from randomization (cumulative risk at 30 days).

To produce an estimation on sample size, different scenarios are considered.

Moreover, due to the to date reduced evidences about COVID-19 infection epidemiology, the sample size estimation could be updated after a one third of population recruited and eventually modified according a substantial protocol amendment.

We first consider an individual randomization design and then adjust for the effect of cluster randomization. According to the individual randomization design, fixing a treated-control ratio 2:1, power of 90%, two-sided probability of type I error at 5%, a reduction of cumulative risk of 25%-30%-35% (Relative Risk =0.75, 0.70 and 0.65 respectively) and a baseline cumulative risk from 2.5% to 30% we have the following detailed results by varying risk in the control group and relative risk (table and figure below). As example a sample size of at least 2000 subjects will assure enough power for RR around 0.70 and 20% risk in the control group.

Risk in the Control group	Expected Risk the Treatment Group	Relative Risk	Size N	N1 (Control)	N2 (Treated)
0.025	0.01625	0.65	12276	4092	8184
0.025	0.0175	0.7	17256	5752	11504
0.025	0.01875	0.75	25629	8543	17086
0.05	0.0325	0.65	6009	2003	4006
0.05	0.035	0.7	8442	2814	5628
0.05	0.0375	0.75	12531	4177	8354
0.1	0.065	0.65	2877	959	1918
0.1	0.07	0.7	4035	1345	2690
0.1	0.075	0.75	5979	1993	3986
0.15	0.0975	0.65	1830	610	1220
0.15	0.105	0.7	2565	855	1710
0.15	0.1125	0.75	3795	1265	2530
0.2	0.13	0.65	1308	436	872

0.2	0.14	0.7	1830	610	1220
0.2	0.15	0.75	2703	901	1802
0.25	0.1625	0.65	996	332	664
0.25	0.175	0.7	1389	463	926
0.25	0.1875	0.75	2049	683	1366
0.3	.195	0.65	786	262	524
0.3	0.21	0.7	1095	365	730
0.3	0.225	0.75	1614	538	1076

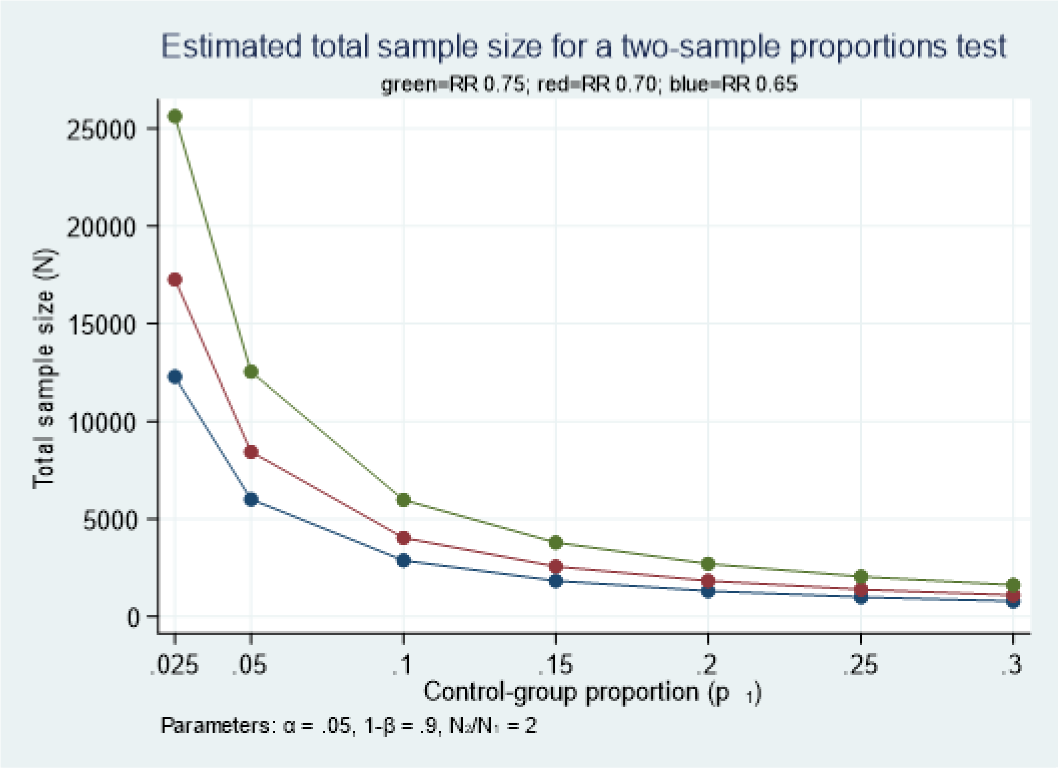

However, the sample size assuming an individual randomization must be inflated by multiplying by the Design Effect, due to the cluster randomization design.

The design effect (Donner and Klar 2000) is: 1+(*n*-1)´r

where *n* is the cluster size and r the Intraclass Correlation Coefficient. The larger the cluster size, the greater the Design Effect.

The Intraclass Correlation Coefficient can be estimated by the ratio of the Between-cluster variance to the sum of Between and Within-cluster variance. For the Between-cluster variance we can use the prevalence on symptomatic patients among areas of residence of the case-patients which identify the clusters. We can approximately use values ranging from 0.15 to 0.30 (average 0.225). A simulation provides estimates of between cluster variance around 0.10. For the Within-cluster variance we can use the probability of getting the disease given being a contact of a case a recent China study [25] reports 0.15 as secondary attack rate. This gives a range of value for the Intraclass Correlation Coefficient up to 0.40.

Conservatively having cluster with variable size, we assume an average *n* around 2 (a plausible alternative would be 1.5).

With regard to the variable cluster size using suggestion from Eldridge and Kerry (2006) [26] we assume a coefficient of variation of the cluster size between 0.7 and 1 (approximately taking as worst scenario a variation in cluster size between 1 and 40).

The design of the study considers also a stratification of the index cases (cluster). The stratification effect is difficult to consider. Theoretically it will deflate the Between-cluster variance but it is prudent to conduct the sample size calculation ignoring the stratification factor (Eldridge and Kerry 2006) [26]. However, if the stratification is successful the Intraclass Correlation Coefficient (ICC) should reduce toward very small values. Therefore, we can take as limit a value for ICC of 0.05.

The figure below shows the more realistic scenarios.

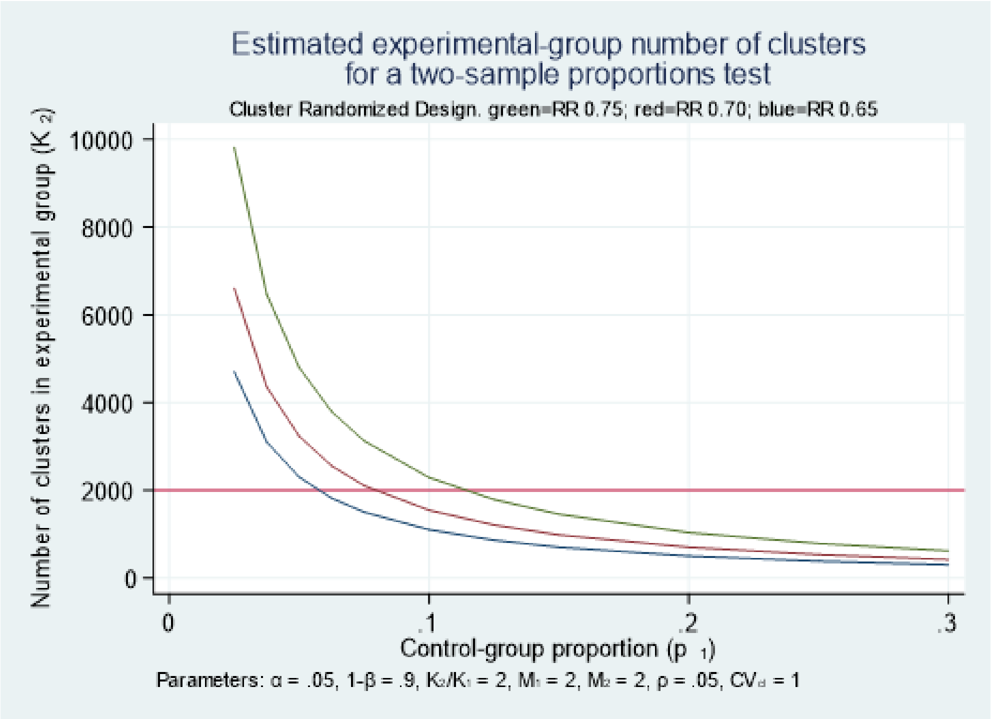

In conclusion we planned to randomize about 2000 subjects of Group 1 and to perform 1 Interim analysis using standard alpha-spending function, we fix α=0.025 for the interim analysis (total type I error probability: 0.025+0.025´0.975=0.05).

Assuming about 1.5-2.0 asymptomatic SARS-CoV-2-exposed subjects and/or contacts for each COVID-19 patient, we expected to identify approximately 1000-1300 COVID-19 index cases.

The stratification will be based on the data collected at cluster level in the preliminary phase of the trial.

Group 2: The primary endpoint/outcome measure is the proportion of subjects of Group 2 who become swab negative (Binary outcome) in each arm within 14 days from randomization (cumulative risk at 14 days).

We will consider eight strata by the 2by2by 2 cross-classification of index-cases (clusters) by:

- COVID-19 risk level for residence (high vs low/intermediate);

- Health care professionals (yes vs no)

- Home situation without COVID-19 treatment (yes vs no)

We estimate on current preliminary data from the Health Information Service of Region Emilia Romagna around 25-30% of positive not-otherwise treated asymptomatic cases (25-30% of about 1000-1300 expected case index COVID-19 patients). However, it is expected an increasing aggressive strategy toward asymptomatic cases, and a percentage of positive not-otherwise treated asymptomatic cases lesser than 30%.

The primary outcome on Group 2 positive not-treated asymptomatic cases is the proportion of negative swab at 14 days from randomization. We assume for the control group a proportion of negative swab between 0.10 and 0.40 while we expect in treatment group a proportion of negative swab between 0.50-0.70.

For Group 2 the sample size is fixed by the choices done for the cluster randomization trials, 30% of the clusters (index cases). We therefore evaluate the power function and we show sufficient power for RR above 1.5 provided the proportion in the control group be greater than 30%, given a sample size of 300 subjects (100 control, 200 treatment arm), figure below.

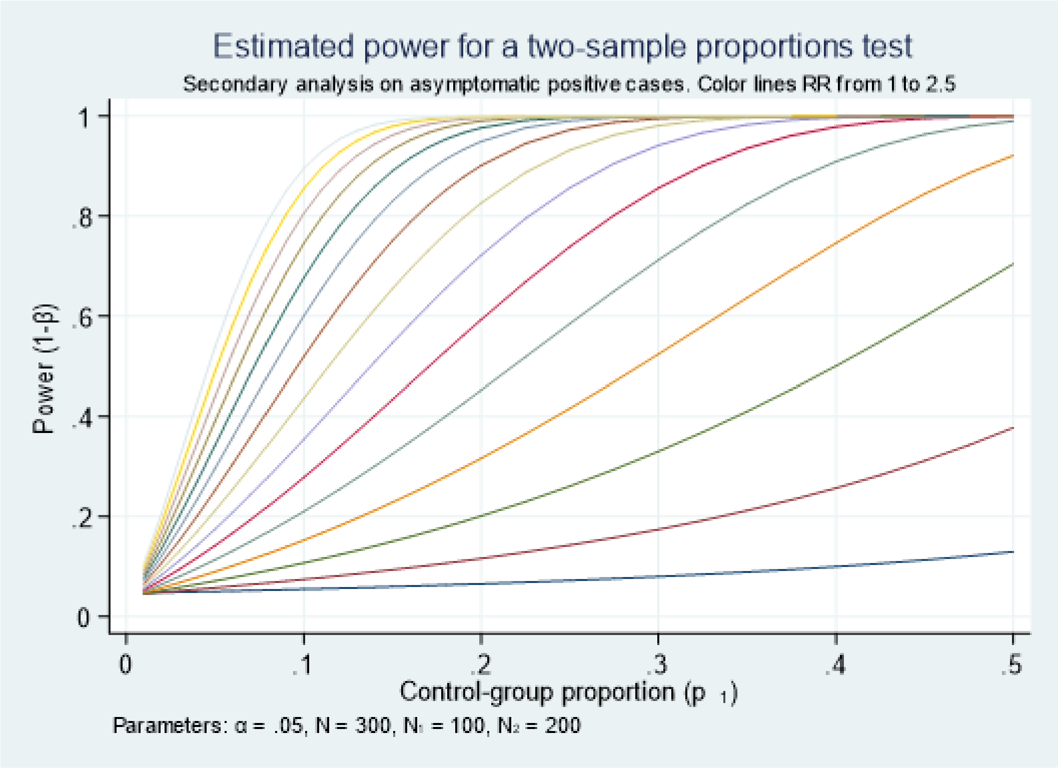

However, due to some uncertainties in the real percentage of clusters (index-cases) which satisfy the inclusion criteria for Group 2 (because of more aggressive strategies on asymptomatic COVID-19 positive cases), we fixed also power to 90% and explore the sample size as function of proportion of negatives in the control group by relative risk between 1.75 and 2.5. Even with less than 300 available positive not-treated asymptomatic cases we can reach power for relative low RR (figure below).

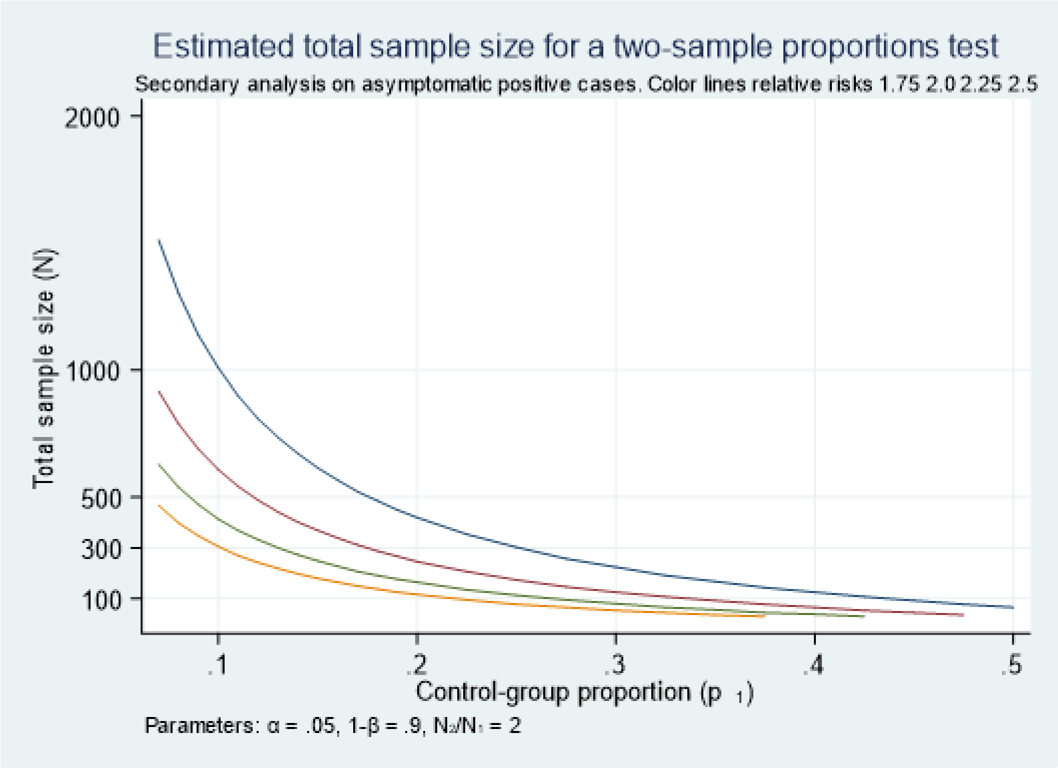

The proportion on negatives in the control group is important on ensuring enough power. With this regard, it should be remembered we are implicitly applying a sequential testing. Therefore, we expect, due to the use of an imperfect test for the diagnosis of an infected patient, that at the second test about ten percent of the positive will score negative. In fact

Pr(negative to second test | positive to first test) = 1 – Pr(positive to the second test | positive to the first) = 1 – PPV(1st test)´Sens/(Pr(positive 2nd test) =

= (0.2´0.9/(0.2´0.9+0.8´0.1)) ´0.9/(((0.2´0.9/(0.2´0.9+0.8´0.1)) ´0.9)+0.1´ (0.2´0.9/(0.2´0.9+0.8´.1))) = 0.10

assuming sensitivity and specificity of 90% and prevalence of 20% at first test. In summary we are confident to find a percentage of negatives in the control group greater that 10%. Interim analysis for this secondary outcome can be performed using the alpha-spending function approach, indicatively considering two interim evaluations. The first interim analysis will be performed at 100 enrolled COVID-19 patients.

The overall study duration will be 12 months; 3 months for subjects enrollment, and 3 months of treatment and further 6 months of follow-up.

10.2 Stratification Factors

Randomization lists will be stratified according to the following factors:

- COVID-19 risk level for residence (high vs low/intermediate);
- Health care professionals (yes vs no)
- Home situation without COVID-19 treatment (yes vs no)

COVID-19 index cases will be randomized (2:1) to either arm A or arm B. An independent statistician not otherwise involved in the trial will generate the allocation sequence, and COVID-19 response teams will be unaware of the allocation of clusters.

10.3 Statistical analysis

We planned a Generalized Estimating Equation analysis to get advantage of subject-specific covariates. This is therefore more efficient than a cluster level analysis. The above reported sample size analysis is therefore to be considered conservative.

10.4 Reporting and Exclusions

All primary and secondary outcomes will be analyzed in ITT population.
The number of patients who are randomized, discontinued, or completed the study will be summarized and reasons for premature study discontinuation will be reported.

11. ETHICAL ASPECTS

This study has been designed and will be performed in a critical emergency situation, determined by the rapid diffusion of the COVID-19. Every effort will be made to ensure that all study procedures will be conducted according to GCP and to european and italian laws regarding clinical trials.

11.1 Declaration of Helsinki

The Investigator will ensure that this study is conducted in full conformity with the current revision of the Declaration of Helsinki (last amended 64th WMA General Assembly, Fortaleza, Brazil, October 2013).

11.2 ICH Guidelines for Good Clinical Practice

The Investigator will ensure that this study is conducted in full conformity with relevant regulations and with the ICH Guidelines for Good Clinical Practice E6 (R2), Regulation (EU) n. 536/2014 of the European

Parliament and other relevant local legislation.

11.3 Independent Ethical Committee (IEC)

The protocol, informed consent and any accompanying material provided to the patient will be submitted by the investigator to the Ethic Committee of the National Institute for Infectious Diseases Lazzaro Spallanzani - IRCCS of Rome, for review, which is the reference committee for trials on COVID-19 in Italy. Approval from the committee must be obtained before starting the study. Any modifications made to the protocol, informed consent or material provided to the patient after receipt of the Ethics Committee approval must also be submitted by the investigator to the Committee in accordance with local procedures and regulatory requirements. The IEC approval report must contain details of the trial (title, protocol number and version), documents evaluated (protocol, informed consent material) and the date of the approval.

11.4 Informed Consent

According to Good Clinical Practice and to European and Italian laws concerning clinical trials, all subjects must personally give written informed consent to participate to a clinical trial before any study procedure. However, given the extraordinary nature of moment due to pandemic diffusion of COVID-19 in Italy and the lockdown imposed by the DPCM 11.03.2020, it is not feasible to provide information to the potentially eligible subjects through a face-to-face interview and to collect a signed informed consent by the patient. As an alternative to written consent, Reg.EU 536, (art 29) allows to record the informed consent through appropriate alternative means when the subject is unable to write. Furthermore, art. 35 “Clinical trials in emergency situations” states that in cases when “due to the urgency of the situation, caused by a sudden life-threatening or other sudden serious medical condition, the subject is unable to provide prior informed consent” it is possible to collect informed consent after the inclusion of patient in the trial.

Taking into account all these considerations, a telephonic procedure to record informed consent is applied in this study, as described in section 5.2. In any case, a signed informed consent form will be obtained by the subjects any time it will be possible.

11.5 Patient data protection

During the telephonic informed consent process the subjects will be informed about relevant data protection and privacy legislation. All enrolled subjects will authorize the collection, use and disclosure of their study data by the Investigator and by those persons who need that information for the purposes of

the study.

During the telephonic informed consent process the subjects will be informed as well that the study data will be stored in a computer database, maintaining confidentiality in accordance with national data legislation.

It will also be explained that for data verification purposes, authorized representatives of Sponsor/Promoter, a regulatory authority, an Ethics Committee may require direct access to parts of records relevant to the study, including patients' medical history.

12. DATA COLLECTION

The coordinating center will prepare and maintain adequate and accurate source documents designed to record all observations and other pertinent data for each subject enrolled.

Study personnel at the coordinating center will enter data into the protocol-specific electronic Case Report Form (eCRF) after each telephonic interview with the subjects. Subjects will not be identified by name in the study database or on any study documents to be collected by the Sponsor/Promoter (or designee), but will be identified by a site number, subject number.

In addition, all subjects will have access to an electronic platform for ePRO (Patient Reported Outcome) collection, which will be available for smartphone or tablet. ePRO will include Quality of life questionnaire EQ-5D-5L and other data (e.g. adverse events, body temperature, onset of COVID-19 symptoms, contacts with COVID-19 patients etc.).

Electronic Case Report Forms

Clinical Reporting Forms (CRF) will be completed through an Electronic Data Capture (EDC). The database will be highly secured and stored in a way that meets all the requirements for data safety and privacy set by international law. All patients' information will be kept private and anonymized. AllCRFs will be completed by designated, trained site staff. eCRFs will be reviewed and electronically signed and dated by the investigator or a designee.

If a correction is required for an eCRF, the time and date stamps track the person entering or updating eCRF data and creates an electronic audit trail.

13. STUDY MONITORING

The Investigators agree to perform the study in accordance with ICH Good Clinical Practice.

The Investigator is required to ensure his compliance to the procedures required by the protocol with respect to the investigational drug schedule and visit schedule. The Investigator agrees to provide all information requested in the Case Report Form in an accurate and legible manner according to the instructions provided.

The Investigator has responsibilities to the Health Authorities to take all reasonable steps to ensure the proper conduct of the study as regards ethics, protocol adherence, integrity and validity of the data recorded on the case report forms.

13.1 Site Set-up and Initiation

All participating Investigators will be asked to sign the necessary agreements and supply a current CV to the coordinating centre or Sponsor/Promoter.

All members of the site research team will also be required to sign the "*Site Signature and Delegation Log*".

Prior to commencing recruitment all sites will undergo a process of initiation. Key members of the site research team will be required to attend either a meeting or a teleconference covering aspects of the trial design, protocol procedures, Adverse Event reporting, collection and reporting of data and record keeping.

Sites will be provided with an Investigator Site File containing essential documentation, instructions, and other documentation required for the conduct of the trial. The coordinating centre or Sponsor/Promoter must be informed immediately of any change in the site research team.

13.2 Study Monitoring

If a monitoring visit is required the coordinating centre, or Sponsor/Promoter will contact the site to arrange a date for the proposed visit and will provide the site with written confirmation. Investigators will allow the trial staff access to source documents as requested.

The main duty of the Trial Monitor is to help the Investigator and the Study Coordinators to maintain a high level of ethical, scientific, technical and regulatory quality in all aspects of the study.

During each monitoring visit, the following points will be checked: subject informed consent, subject recruitment and follow-up, study drug allocation, subject compliance to the study treatment, study treatment accountability, Adverse Event documentation and reporting.

According to the guidelines on ICH Good Clinical Practice, the trial monitor will check the case report form

entries against the source documents. This personnel, bound by professional secrecy, will not disclose any personal identity or personal medical information.

13.3 Central Monitoring

Trials staff will be in regular contact with the site research team to check on progress and address any queries that they may have. Trials staff will check data received for compliance with the protocol, data consistency, missing data and timing. Sites will be sent requests missing data or clarification of inconsistencies or discrepancies. For eCRF trials these requests may be generated by automated data validation checks.

13.4 Independent Data Monitoring Committee

The trial staff will organize periodic meetings during the study withIDMC to discuss the study progress and review the safety and efficacy data. The IDMC will be in charge of reviewing the data at the interim analysis and at final analysis and will draft a report that will be discussed with the scientific committee.

13.5 Audit and Inspection

The Investigator will permit trial-related monitoring, audits, ethical review, and regulatory inspection(s) at their site, providing direct access to source data/documents.
Sites are also requested to notify the coordinating centre or Sponsor/Promoter of any CA inspections.

14. ADMINISTRATIVE REGULATIONS

The CC is responsible for drawing up the final version of the protocol, implementing the CRFs, creating randomization lists and updating the electronic database, defining general organizational procedures, maintaining contact with the IDMC and organizing periodic meetings and newsletters. The CC will also undertake the following: support for the preparation of all documents needed for EC submission of the study protocol for each participating center, training of staff assigned to data collection, definition of monitoring procedures and monitor training.

14.1 Curriculum vitae

An updated copy of the curriculum vitae of each Principal Investigator, duly signed and dated, will be provided to the study monitor prior to the beginning of the study.

14.2 Secrecy agreement

All goods, materials, information (oral or written) and unpublished documentation provided to the Investigators, including this protocol and the case report forms, shall be considered confidential and may not given or disclosed to third parties.

14.3 Availability and Retention of Investigational Records

The Investigator must make study data accessible to the monitor, other authorized representatives of the Sponsor/Promoter (or designee), IEC, and Regulatory Agency inspectors upon request. A file for each subject must be maintained that includes the signed Informed Consent and copies of all source documentation related to that subject. The Investigator must ensure the reliability and availability of source documents from which the information on the CRF was derived.

All study documents (patient files, signed informed consent forms, copies of CRFs, Study File Notebook, etc.) must be kept secured for a period of 15 years after completion or discontinuation of the study or for the length of time required by relevant national or local health authorities, whichever is longer.There may be other circumstances for which the Sponsor/Promoter is required to maintain study records and, therefore, the Sponsor/Promoter should be contacted prior to removing study records for any reason.

14.4 Insurance

Regarding insurance, since this is a non-profit study, the contents of chapter 6 of article 40 of Legislative Decree 23 of the 8th of April 2020 applies "For the non-profit experimental studies referred to in this article, the stipulation of a specific insurance policy is not required" and IRST declares that, as promoter and sole participating institution, it provides suitable insurance coverage for the study.

15. OWNERSHIP OF DATA AND USE OF THE STUDY RESULTS

The full ownership of the data generated in this study is retained by IRST and by all the investigators actively recruiting patients.

Data deriving from this clinical trial are not intended for drug registration or for patent applications, but only for scientific and educational purposes, which include presentation at scientific meetings, congresses and symposia and/or publication in scientific journals.

16. PUBLICATION POLICY

Publications regarding the main study end-points will be prepared by the members of the Scientific

Committee. Authorship will be proportional to the accrual of each center. All the members of the scientific committee and all persons giving substantial contribution to the study will be included in the authors list and all the investigators recruiting will be mentioned as contributors. Other area-specific publications will be prepared by the coordinators of the single treatment modalities to increase the visibility of the study and investigators. However, the publication of secondary endpoints is discouraged before publication of the main endpoint and should be anyway discussed with the study and writing committee coordinators.

17. PROTOCOL AMENDMENTS

It is specified that the appendices, attached to this protocol and referred to in the main text of this protocol, form an integral part of the protocol.

No changes or amendments to this protocol may be made by the Investigators after the protocol has been agreed to and signed by both parties .Any change agreed upon will be recorded in writing, the written amendment will be signed by the Chief Investigator and by the Principal Investigator and the signed amendment will be appended to this protocol.

Approval / advice of amendments by Ethical Committees or similar body is required prior to their implementation, unless there are overriding safety reasons.

If the change or deviation increases risk to the study population, or adversely affects the validity of the clinical investigation or the subject's rights, full approval / advice must be obtained prior to implementation. For changes that do not involve increased risk or affect the validity of the investigation or the subject's rights, approval / advice may be obtained by expedited review, where applicable.

In some instances, an amendment may require a change to a consent form. The Investigator must receive approval / advice of the revised consent form prior to implementation of the change.

18. REFERENCES

1- Na Zhu, Ph.D., Dingyu Zhang, M.D., Wenling Wang, Ph.D., et al.; A Novel Coronavirus from Patients with Pneumonia in China, 2019. February 20, 2020. N Engl J Med 2020; 382:727-733 DOI: 10.1056/NEJMoa2001017

2- https://www.iss.it/

3- Symptoms of Novel Coronavirus (2019-nCoV) – CDC. https://www.cdc.gov/coronavirus/2019-ncov/about/symptoms.html

4- Xiaoling Xu, Mingfeng Han, Tiantian Li, et al.; Effective Treatment of Severe COVID-19 Patients with Tocilizumab. chinaXiv:202003.00026v1

5- Hollander JE, Carr BG. Virtually perfect? Telemedicine for Covid-19. N Engl J Med 2020 225 Mar 11

6- Graziano Onder, MD, PhD; Giovanni Rezza, MD; Silvio Brusaferro, MD; Case-Fatality Rate and Characteristics of Patients Dying in Relation to COVID-19 in Italy. March 23, 2020

7- http://www.irst.emr.it/

8- Home care for patients with COVID-19 presenting with mild symptoms and management of their contacts. World Health Organization. Interim guidance 17 March 2020

9- Considerations for quarantine of individuals in the context of containment for coronavirus disease (COVID-19). World Health Organization. Interim guidance 19 March 2020

10- Wang M, Cao R, Zhang L, Yang X, Liu J, Xu M, Shi Z, Hu Z, Zhong W, Xiao G. Remdesivir and chloroquine effectively inhibit the recently emerged novel coronavirus (2019-nCoV) in vitro. 2020 Mar;30. DOI: 10.1038/s41422-020-0282-0

11- Gautret P, Lagier J, Parola P, et al. Hydroxychloroquine and azithromycin as a treatment of COVID-19: results of an open-label non-randomized clinical trial. Int J Antimicrob Agents 2020 Mar 20. [Epub ahead of print] PMID: 32205204

12- Zhonghua Jie He He Hu Xi Za Zhi. Expert consensus on chloroquine phosphate for the treatment of novel coronavirus pneumonia]. 2020 Mar 12;43(3):185-188. doi: 10.3760/cma.j.issn.1001-0939.2020.03.009

13- Liu, J., Cao, R., Xu, M. *et al.* Hydroxychloroquine, a less toxic derivative of chloroquine, is effective in inhibiting SARS-CoV-2 infection in vitro. *Cell Discov* 6, 16 (2020). https://doi.org/10.1038/s41421-020-0156-0

14- World Health Organization (WHO). Coronavirus: landscape analysis of therapeutics as of 17 Februrary 2020. Accessed March 16, 2020. Available on the World Wide Web at Updated March 26, 2020 Elsevier © 2020 https://www.who.int/blueprint/.

15- Cortegiani A, Ingoglia G, Ippolito M, et al. A systematic review on the efficacy and safety of chloroquine for the treatment of COVID-19. J Crit Care 2020 Mar 10. [Epub ahead of print] PMID: 32173110

16- Plaquenil (hydroxychloroquine) package insert. St. Michael, Barbados: Concordia Pharmaceuticals, Inc.; 2017 Jan.

17- Credible Meds. COVID-19 experimental therapies and TdP risk. Retrieved March 24, 2020. Available on the World Wide Web at: https://crediblemeds.org/blog/covid-19-experimentaltherapies-and-tdp-risk/

18- Adeyemi O. Adedeji, William Severson, Colleen Jonsson, Kamalendra Singh, Susan R. Weiss, Stefan G. Sarafianosa; Novel Inhibitors of Severe Acute Respiratory Syndrome Coronavirus Entry That Act by Three Distinct Mechanisms. J Virol. 2013 Jul; 87(14): 8017–8028. doi: 10.1128/JVI.00998-13

19- Adeline Heurich, Heike Hofmann-Winkler, Stefanie Gierer, Thomas Liepold, Olaf Jahn, Stefan Pöhlmann; TMPRSS2 and ADAM17 Cleave ACE2 Differentially and Only Proteolysis by TMPRSS2 Augments Entry Driven by the Severe Acute Respiratory Syndrome Coronavirus Spike Protein. DOI: 10.1128/JVI.02202-13

20- John E. Kearney, B.S., Chloroquine as a Potential Treatment and Prevention Measure for the 2019 Novel Coronavirus: A Review. 2020 March. doi:10.20944/preprints202003.0275.v1

21- Siddiqu HK, Mehra MR. COVID-19 Illness in Native and Immunosuppressed States: A ClinicalTherapeutic Staging Proposal. Journal of Heart and Lung Transplantation. doi: 10.1016/j.healun.2020.03.012

22- PujaMehta, Daniel FMcAuley, MichaelBrown, EmilieSanchez, Rachel STattersall, Jessica JManson, HLH Across Speciality Collaboration, UK. COVID-19: consider cytokine storm sindrome and immunosuppression. https://doi.org/10.1016/S0140-6736(20)30628-0

23- Gautret et al. (2020) Hydroxychloroquine and azithromycin as a treatment of COVID-19: results of an open-label non-randomized clinical trial. International Journal of Antimicrobial Agents – In Press 17 March 2020 – DOI: 10.1016/j.ijantimicag.2020.105949

24 -Tan KR, Mali S, Arguin PM (2010). "Malaria Risk Information and Prophylaxis, by Country". Travelers' Health - Yellow Book. Centers for Disease Control and Prevention. Retrieved 20 December 2010

25 Stephen A, Lauren, MS, Kyra H, Grantz BA Qifang BI, et al. The incubation period of coronavirus Disease 2019 (COVI-19) from publicly reported confirmed cases: estimation and Application. Annals of Internal Medicine. Pub date: 2020-03-10 DOI: 10.7326/m20-0504

26- Eldridge SM, Ashby D, Kerry S. Sample size for cluster randomized trials: effect of coefficient of variation of cluster size and analysis method. Int J Epidemiol 2006 Oct;35(5):1292-300. DOI: 10.1093/ije/dyl129

APPENDIX ANCI Common Terminology Criteria for AE

See https://ctep.cancer.gov/protocolDevelopment/electronic_applications/ctc.htm#ctc_50

APPENDIX B Schedule of procedures

Period/ Procedure	Screening and registration	1st month	Follow up
**Study Day/Visit Day**		**Every week (+/- 3days)**	**Every 4 weeks (+/- 3days)**
Informed consent[a]	X		
Demographic[b]	X		
Medical history[c]	X		
History of COVID-19 contacts[d]	X		
Concomitant medications	X	X	X
Health status[e]	X	X	
Body temperature[f]	X	X	
COVID-19 symptoms assessment[g]	X	X	X
QoL (EQ-5D-5L)	X	X	X
AE		X	X
Rhino/Oro-pharyngeal swab/assay result[h]		(X)[i]	(X)
Hydroxychloroquine administration[j]		X	
Blood sampling for explorative objective[k]		(X)	(X)

NOTES:

[a]Informed consent will be recorded during telephonic interview with appropriate instruments. The physician conducting the interview will fill in a specific form with subject's answers, print and sign the form and one copy will be sent to the participant by mail. Whenever possible, an informed consent signed by the subject in person will be collected.
[b]Age, sex, ethnic origin, city of residence
[c]Medical history includes: comorbidities present at baseline; flu vaccine history; history of recent surgical interventions; history of cancer and of any chemo/radiotherapy in the previous 6 months; history of respiratory, cardiovascular, allergic, infectious, inflammatory, autoimmune diseases, G6PDH deficiency; any immunosuppressive or antiinflammatory therapy in the previous 6 months; most recent blood exams results if available (blood group, complete blood count, glucose, PCR, liver and kidney functionality, coagulation panel)
[d]date, type and frequency of contact with any COVID-19 patient, known asymptomatic SARS-CoV-2 positive subject, subjects with any symptoms compatible with COVID-19
[e]a list of questions will be asked to patients, regarding evaluation of his/her health status
[f]any change in body temperature will be recorded
[g]COVID-19 symptoms include fever, coughing, sore throat and shortness of breath, disgeusia, diarrhea, vomit
[h]Any time a subject undergoes rhinopharyngeal swab for SARS-CoV-2 diagnostic assay, the date and result of the test will be recorded.
[i] Rhino/oro-pharyngeal swab will be performed to subjects of group 2 after 2 and 4 weeks from study entry
[j]Only for subjects randomized to ARM A, daily dose, dose interruption, reason for dose interruption (patient error, AE, other)
[k]Blood sampling for explorative objectives will be performed whenever it will be possible, at baseline, during treatment or at follow up, either at subject's home or at IRST IRCCS/AUSL of Romagna (after end of quarantine/lockdown period) . A specific informed consent will be obtained before sample collection.

APPENDIX C World Medical Association Declaration of Helsinki

The current Declaration of Helsinki can be found on the World Medical Association web page via the link provided below:

http://www.wma.net
